# Synthesis of Water-Dispersible Poly(dimethylsiloxane) and Its Potential Application in the Paper Coating Industry as an Alternative for PFAS-Coated Paper and Single-Use Plastics

**DOI:** 10.3390/polym16071006

**Published:** 2024-04-07

**Authors:** Syeda Shamila Hamdani, Hazem M. Elkholy, Alexandra Alford, Kang Jackson, Muhammad Naveed, Ian Wyman, Yun Wang, Kecheng Li, Syed W. Haider, Muhammad Rabnawaz

**Affiliations:** 1 School of Packaging, Michigan State University, 448 Wilson Road, East Lansing, MI 48824, USA; hamdanis@msu.edu (S.S.H.); elkholyh@msu.edu (H.M.E.);; 2 Department of Chemical and Paper Engineering, Western Michigan University, 1903 W, Michigan Avenue, Kalamazoo, MI 49008, USA; 3 Department of Civil & Environmental Engineering, Michigan State University, East Lansing, MI 48824, USA

**Keywords:** biodegradable, PFAS alternatives, waterborne coatings, water-resistant, oil-resistant, pulp recovery

## Abstract

Polyethylene-, polyvinylidene chloride-, and per- and polyfluoroalkyl substance-coated paper generate microplastics or fluorochemicals in the environment. Here, we report an approach for the development of oil-resistant papers using an environmentally friendly, fluorine-free, water-dispersible poly(dimethylsiloxane) (PDMS) coating on kraft paper. Carboxylic-functionalized PDMS (PDMS-COOH) was synthesized and subsequently neutralized with ammonium bicarbonate to obtain a waterborne emulsion, which was then coated onto kraft paper. The water resistance of the coated paper was determined via Cobb60 measurements. The Cobb60 value was reduced to 2.70 ± 0.14 g/m^2^ as compared to 87.6 ± 5.1 g/m^2^ for uncoated paper, suggesting a remarkable improvement in water resistance. Similarly, oil resistance was found to be 12/12 on the kit test scale versus 0/12 for uncoated paper. In addition, the coated paper retained 70–90% of its inherent mechanical properties, and more importantly, the coated paper was recycled via pulp recovery using a standard protocol with a 91.1% yield.

## 1. Introduction

The packaging industry’s reliance on single-use plastic and plastic-coated paper has become a major concern for our modern society as these materials end up in landfills as municipal solid waste, and they often leak into the oceans and other bodies of water, where they are converted into small particles known as microplastics [1,2,3,4]. These single-use plastics [5], as well as plastic-coated paper, are the leading cause of the accumulation of plastics and microplastics in the environment [6], and this accumulation has increased radically in recent years [7,8]. There are approximately 150 million tons of plastic floating in marine environments along with an additional dose of approximately 8 million tons of plastic coming each year to the oceans. In response to these concerns, Europe has supported banning the use of single-use plastics by 2021 [9,10]. Also, some states in the US have banned some single-use plastic products [11,12].

Per- and polyfluoroalkyl substances (PFASs) have found various applications in the paper industry [13,14,15] for the manufacturing of various paper-based materials, including plates, cups, and boxes, because they offer desirable mechanical properties along with other features such as low weight, food-safe nature, and extensive accessibility [16,17]. However, the structure of paper contains polar hydroxyl groups, and in unmodified form, its porous structure is very prominent, which is the main cause of poor barrier properties of paper as well as water and oil resistance [18,19,20]. Numerous studies were carried out over the years to develop water- and oil-resistant paper products, and different strategies were picked to overcome poor barrier properties of paper products, including physical and chemical modification of the paper surface [21], application of coating material [22], and paper sizing [23]. Among these, the most common approaches for bringing water and oil resistance are to laminate paper with plastics (e.g., polyethylene and polyvinylidene chloride) or to coat paper substrates with low-surface-energy chemicals such as PFASs [24,25]. These modified paper substrates have limited recyclability because of associated challenges with the collection, sortation, and removal of applied plastic coating from paper substrate. Due to these problems, these paper products often go to landfills after usage, which subsequently results in leakage to marine ecosystems [26,27]. Paper should be oil-resistant to be suitable for food packaging applications such as disposable paper plates, pizza boxes, paper cups, and fast-food wrappers. Accordingly, paper substrates are often imparted with these attributes by coating them with low-surface-energy PFAS compounds [28]. Consequently, fluorine-modified paper is used in many packaging products, including, but not limited to, baking and sandwich wrappers, paperboards, and paper baking forms [14]. However, fluorinated chemicals are a source of long-lasting damage to the environment, and some of them are toxic [29]. The presence of fluorochemicals in paper products has led to concerns regarding potential exposure during the production, use, and disposal of these materials [30]. Epidemiological analysis has revealed a possible connection between human contact with the long-chain PFAS compounds perfluorooctanesulfonic acid (PFOS) and perfluorooctanoic acid (PFOA) for a long time and certain diseases like kidney disease, thyroid disease, abnormal birth, male reproductive issues, issues related to pregnancy, and immune system dysfunction in children [31,32,33,34,35]. In August 2018, the state of Michigan declared emergencies in two towns due to the presence of fluorinated materials in underground water at concentrations exceeding the permissible limits of 70 parts per trillion by 20-fold [36]. Considering the environmental challenges associated with the use of PFASs for paper coatings and microplastics originating from single-use plastics, environmentally benign processes for water- and grease-resistant paper are urgently needed. Our group has developed water- and oil-resistant coated paper using polydimethylsiloxane (PDMS)–melamine [3,36,37], chitosan-grafted-PDMS, a chitosan–zein dual-layer approach [38], starch- and zein-based coated paper [39], a polyvinyl alcohol- and zein-based bilayer approach [23], and biopolymer-based high barrier properties [18,40,41,42]. A significant advantage of this work compared to prior PDMS studies is the use of waterborne coatings without relying on harmful chemicals. Unlike the previous research, which utilized isocyanates as linkers for waterborne coatings, this study avoids such hazardous substances.

The focus of this research is to develop water-dispersible PDMS and evaluate its application in the paper coating industry as an alternative to PFAS-coated paper. The paper industry employs both melt and water dispersion methods as they offer both worker and food safety. Of course, if solvent residues are present, they can migrate to food. Waterborne coatings are superior to melt coating for certain applications, particularly for coating 3D paper objects like fiber trays, where melt coatings are constrained to 2D surfaces. Consequently, our study aimed to develop a waterborne coating suitable for coating both 2D and 3D fiber/paper articles. For this purpose, first, carboxyl-functionalized PDMS (PDMS-COOH) was synthesized. The obtained PDMS-COOH was dispersed in water along with starch and then coated onto kraft paper. Subsequently, the water and oil resistance properties and mechanical properties of coated paper samples were investigated.

## 2. Experimental Section

### 2.1. Materials

Unbleached kraft paper with a basis weight of 137 ± 0.5 g/m^2^ was purchased from Uline (Pleasant Prairie, WI, USA) and used as substrate. Corn starch was purchased from Aldrich (St. Louis, MO, USA). Carbinol (hydroxyl)-terminated polydimethylsiloxane 50–65 cSt (DMS-C16 (*M*_w_ = 600–850 g/mol) was purchased from Gelest. *Meso*-butane-1,2,3,4-tetracarboxylic dianhydride (MBTCA) was purchased from TCI AMERICA (Portland, OR, USA). Tin(II) 2-ethylhexanoate and ammonium bicarbonate were bought from Sigma Aldrich. There was no prior modification or purification conducted for any chemical before its use.

### 2.2. Methods

#### 2.2.1. Synthesis of Carboxylic-Functionalized PDMS (PDMS-COOH)

A single-neck round-bottle flask was charged with DMS-C16 (1 mole equiv., 70 g), MBTCA (1 mole equiv., 16.31 g), and tin-2-ethylhexanoate (0.5 wt% with respect to PDMS). The resultant mixture was stirred at 170 °C for 1 h under an ambient atmosphere to obtain the desired product PDMS-COOH without using any solvent. In the beginning, the reaction mixture was completely liquid, and the viscosity started to increase with time. After 30 min, the reaction mixture was too viscous, and the reaction continued for a further 30 min. Based on the ^1^H-NMR spectrum, the reaction conversion was 97%.

**Figure 1:**
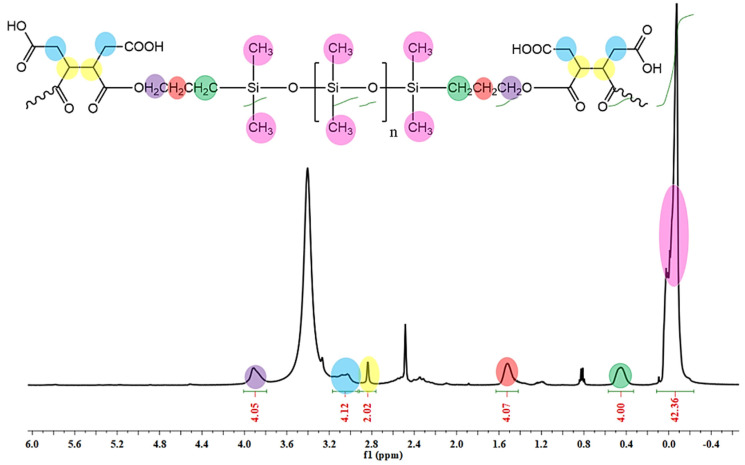
^1^H-NMR spectrum of PDMS-COOH.

^1^H-NMR (DMSO-*d*_6_) (Figure 1): δ (ppm) = −0.07–0.02 (*b*, CH_3_-Si), 0.46 (*b*, 4H, CH_2_-Si), 1.52 (*b*, 4H, CH_2_-CH_2_-CH_2_), 2.84 (*b*, 2H, CHCOO), 3.03–3.26 (*b*, 4H, CH_2_COOH), 3.92 (*b*, 4H, CH_2_-OCO).

#### 2.2.2. Emulsification of PDMS-COOH

A weighed amount of PDMS-COOH was dissolved in an aqueous solution of ammonium bicarbonate (two mole equivalents with respect to the MBTCA molecules in 5 mL of deionized water). The pH of the final emulsion was 7~8.

#### 2.2.3. Preparation of Starch Solution

A stock solution of 5% starch was prepared for making base paper by dissolving starch in deionized water, using a 200 mL beaker covered with aluminum foil at 100 °C, which was stirred until a homogeneous translucent solution was obtained.

#### 2.2.4. Preparation of Starch and PDMS-COOH Mixture Solution

First, starch of the desired weight (g) was dissolved in 5 mL of water and heated at 100 °C on stirring for 10–15 min until a clear homogeneous solution was obtained. Then, it was blended with an emulsion of PDMS-COOH using a vortex mixer to obtain the final coating solution according to the different formulations shown in Table 1.

#### 2.2.5. Paper Coating Procedure

Starch-coated kraft paper samples were prepared by applying 5% starch solution onto uncoated kraft paper using a rod coater K303 Multi Coater, purchased from RK Print Coat Instruments (New Castle, DE, USA), followed by air drying for 24 h. Prior to the application of the designed coating solution, 5% starch-coated kraft paper was trimmed into 20 × 15 cm^2^ sections and fixed to aluminum plates with tape. The coating material was then applied using a silicon spatula following the doctor blade method. The resultant coated paper substrates were dried in a preheated oven at 130 °C for 30–40 min. This coating procedure was performed in batches to ensure the application of the coating in a smooth and uniform manner. After they had been dried in an oven, all the samples were air dried for 24 h prior to testing and further analysis. The final formulations used in this study and their corresponding codes of coated papers are provided in Table 1. The amount of PDMS-COOH, starch, and ammonium bicarbonate used in grams is shown in Table S1.

## 3. Characterization

### 3.1. ATR-FTIR Analysis

A Jasco FTIR-6600 spectrometer (Easton, MD, USA) was employed to record the FTIR spectra of paper samples as well as solid materials used for coating. To obtain a clear signal-to-noise ratio, the spectra were recorded over the wavelength range of 4000–500 cm^−1^ with a total number of 32 scans.

### 3.2. ^1^H-NMR Analysis

Each polymeric sample was characterized by ^1^H NMR spectroscopy (500 MHz, Varian 7600-AS, Palo Alto, CA, USA). The sample was prepared by dissolving 5 mg of the respective polymer in 0.7 mL of dimethyl sulfoxide-*d_6_*.

### 3.3. Basis Weight and Thickness

A digital micrometer (Testing Machine Inc., New Castle, DE, USA) was used for recording the thickness of paper samples. The thickness of all the samples was recorded in μm from ten different spots of paper samples and was reported as an average value.

The basis weight was recorded as the mass per square meter following the standard protocol [43], taking a sample of size 10 × 5 cm^2^. The paper sample was weighed before the coating process, followed by coating. Once the sample was completely dry, it was weighed again to find the basis weight on the difference of weight. Equation (1) was used for calculating the value of basis weight.

Basis weight = weight (g)/area (m^2^) (1)

The coating load of coated paper samples was determined via Equation (2), which is the difference in basis weight before and after the coating.

Coating Load = basis weight (coated paper − uncoated paper)(2)

The % increase in coating load was established by calculating coating load by wt% using a paper sample of size 10 × 5 cm^2^ and following Equation (3). The difference between the weights of coated and uncoated paper was recorded in g, and then this difference was divided by the weight of uncoated paper. Subsequently, the resultant value was multiplied by 100.

Coating Load (wt%) = [weight after coating − weight before coating/weight of uncoated paper] × 100(3)

Difference = Weight of coated paper (g) − weight of uncoated paper (g)]

### 3.4. Scanning Electron Microscopy (SEM)/Energy-Dispersive X-ray Spectroscopy (EDX)

A JEOL 6610 SEM system (JEOL Ltd., Tokyo, Japan) was used for SEM analysis. Prior to analysis, a thin gold layer (15 nm) was applied to the paper samples employing the sputtering technique. The surface morphology was explored to find a difference before and after coating on the surface of paper samples. Energy-dispersive X-ray spectroscopy (elemental analysis) was carried out using an Oxford Instruments Aztec system (Oxford Instruments, High Wycombe, Bucks, UK). The software version 3.1 was used along with a 20 mm^2^ Silicon Drift Detector (JEOL 6610LV SEM) and an ultra-thin window.

### 3.5. Water Resistance

The water resistance of the coated paper samples was analyzed in comparison to uncoated paper used as a control, following a standard Cobb tests [44], which were performed for 60 s. The paper samples were trimmed to a size of 100 cm^2^, and the Cobb60 value was measured using a Cobb sizing tester (Büchel BV Inc., Utrecht, The Netherlands). A volume of 100 mL of deionized water (DI) water was placed in contact with the paper samples for 60 s. The samples were weighed before and after the test, and the difference was recorded as a Cobb value. The results were expressed in units of grams per square meter (g/m^2^).

To evaluate the water resistance against liquid water, the droplet behavior test was performed by applying a droplet with a volume of 0.1 mL onto paper samples to visualize the water absorption. Time was counted, and images were taken at different time intervals, before applying the droplet, after applying the droplet for 5 min, and after removal of the droplet to see penetration of the droplet on the surface of coated paper.

### 3.6. Water Vapor Transmission Measurements

The resistance of water was also recorded against water vapor by measuring the water vapor transmittance rate (WVTR) in parallel to the liquid water resistance to evaluate the barrier properties of coated paper samples. The WVTR was recorded using a Permatran-W system (Model 3/34, Mocon Inc., Minneapolis, MN, USA). The samples were prepared by fixing paper samples with dimensions of 2 × 2 cm^2^ in an aluminum mask sheet. The sample was exposed to water vapors by leaving an open hole with an area of 0.5 cm^2^. All the tests were performed at 23 °C and at 50% relative humidity (RH). The samples were preconditioned at these desired parameters for one hour prior to recording their analysis inside the machine.

### 3.7. Oil Resistance Properties

The Standard protocol [45] was followed to measure the oil or grease resistance of paper samples. For this analysis, a series of kit test solutions (Nos. 1–12) having different ratios of castor oil, heptane, and toluene [3] was prepared. The kit test results were expressed in numbers ranging from 1 to 12. Kit number 1 corresponds to less oil resistance, while number 12 indicates the highest oil resistance. A liquid droplet with a volume of 0.05 mL was placed on each paper sample for 15 s and then wiped away using chem wipes. The surface under testing was visualized, and it was considered to have failed the test performed with the liquid of a given kit number if any dark spot appeared following the application and removal of that liquid. The absence of any dark spots following the application and removal of a liquid with a given kit number indicated that the sample had passed the test performed at that kit number. From these tests, the kit rating of a given sample corresponded to the highest kit number of the liquid that could be applied to the sample and removed without leaving any spots. The oil droplet behavior study was carried out in a similar manner to the water droplet test, except that neat castor oil was employed instead of water.

### 3.8. Thermogravimetric Analysis (TGA)

The thermal stability of coated paper samples after application of the coating was analyzed by TGA. A Q-50 thermogravimetric analyzer (TA Instruments, New Castle, DE, USA) was used for this analysis, and measurements were taken over a temperature range of 10–600 °C. Samples with a weight of 8 mg were each heated in a standard pan at a ramping rate of 10 °C/min. The test was performed under a nitrogen atmosphere at a flow rate of 40 mL/min. The derivative thermogravimetric (DTG) curves were recorded by studying the first derivative of the TGA curves.

### 3.9. Contact Angle Measurements

To determine the contact angles (CAs), 5 µL droplets were placed on the surface of coated and non-coated paper specimens. A 590-U1 AST VCA 2500XE Video Contact Surface Inspection Goniometer Fuji 611847 (AST Products, Inc., Billerica, MA, USA) was used to record images of the droplets and to determine the CAs after intervals of 30 s and 5 min of the application of the droplets. The result was reported as the mean value of the recorded CAs. Water and castor oil were used as test liquids for measurements of the water and oil CAs, respectively.

### 3.10. Tensile Measurements

The tensile properties of the coated paper sample were explored using a 5565 Universal Instron Testing Machine (Instron, Norwood, MA, USA), following the standard protocol [46]. Paper samples, each of which had specific dimensions of 1 × 4 inches^2^, were made using a JDC precision sampler cutter. All the samples were investigated while maintaining a gap separation of 25 mm and stretched at a constant rate of 12.5 mm/min. The tensile properties were recorded using Bluehill software Universal (Instron, Norwood, MA, USA).

### 3.11. Ring Crush Test (RCT)

Ring crush test (RCT) measurements give an idea about the resistance of a paper ring with a specific length and width. The ring crush resistance was determined using a special TMI crush tester. A standard protocol [47], was followed to investigate RCT value using Emerson’s Model 1210 Crush Tester (Boston, MA, USA) in both the cross direction (CD) and machine direction (MD). All the results were recorded in triplicates, and an average value has been reported.

### 3.12. Bending Stiffness (BS)

The measurement of bending stiffness (BS) was performed following the T standard protocol [48]. The instrument used was a Taber Stiffness Tester (Model 150-D, Teledyne Taber, North Tonawanda, NY, USA), employing a force of 1000 Taber stiffness units. The prepared paper samples with dimensions of 1.5 × 2.5 inches^2^ were fixed between the clamps of the instrument and readings were obtained by bending sample upto 15° in both the right and left directions. The mean value of the reading obtained in right and left bending was noted, and the BS was calculated by multiplying the average value by the P number, where P is equal to 10 when a force of 1000 Taber stiffness units is exerted on the edges of the paper.

### 3.13. Internal Tearing Resistance (ITR)

The internal tearing resistance (ITR) of coated paper samples in comparison to uncoated paper was recorded with the use of the ME-1600 Manual Elmendorf-Tearing Tester (Oakland Instrument Co., Shakopee, MN, USA). The standard procedure [49] was followed for analysis. Two piles of the same sample were fixed between knobs of the instrument, and the average value of internal tearing force was recorded in grams using Equation (4).

Internal tearing force, (g) = (16 × scale reading)/number of plies of sample used(4)

The strength of the fiber was determined by measuring the burst strength using a Messmer Büchel (Fokkerstraat, The Netherlands) burst tester. Samples with dimensions of 14 × 14 cm^2^ were used, and the results were recorded in psi.

### 3.14. Recyclability

The repulping approach reported by our group [1] was used for recycling coated paper. The coated paper (3 g) was finely chopped into small pieces with a size of 3 cm × 3 cm and subsequently immersed in warm water (70 °C) for one hour to remove the starch coating. The paper that had been soaked for one hour was then put into a blender to obtain paper pulp. The pulp was passed through a wooden screen to remove water and the dissolved starch coating. The obtained pulp was washed with ammonium bicarbonate solution (2%) using a centrifuge. The washing was repeated in triplicate to remove all the coating material by removing the supernatant. The washed pulp was converted to recycled paper by pressing it with an iron, followed by complete drying in a preheated vacuum oven at 60 °C for four hours. ATR-FTIR analysis of recycled paper (P4S1-NH_4_) was performed to confirm the separation of pulp from the coating material. The sample B-KP was washed in a similar manner with ammonium bicarbonate to see the effect of washing on neat pulp.

To investigate the standard repulpability of PDMS-COOH-coated paper, the FBA voluntary standard for repulping and recycling corrugated fiberboard protocol was used [50]. A 25 g sample of the selected coated paper (P1S0), which was mainly coated with PDMS-COOH emulsion, was trimmed down to small pieces with dimensions of 1 × 4 inches^2^. The trimmed pieces were soaked in 1500 mL of warm water at 52 °C for 4 h. The soaked paper was transferred to a preheated blender and blended at a speed of 15,000 rpm for four minutes prior to deflaking for five minutes in a British disintegrator of 2000 mL total volume at 3000 rpm and 52 °C.

The obtained slurry was filtrated using a 0.010-inch slotted open screen for 20 min. The pulped material was separated and dried in a laboratory oven at 105 °C for 12 h to determine the fiber recovery as a percentage of the amount of fiber charged.

The yield of repulping was calculated via Equation (5):

(5) $$Yield\%=\frac{Screen\;Accepted}{Screen\;Accepted+Net\;Rejected}\times 100$$

## 4. Results and Discussion

**Scheme 1:**
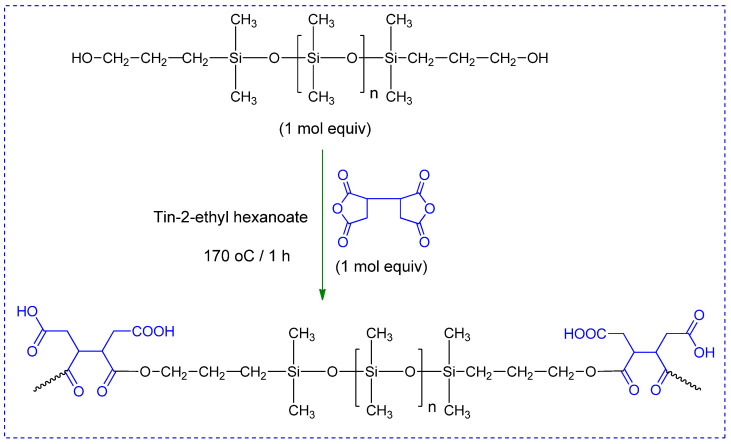
Illustration of the synthetic route leading to PDMS-COOH.

To produce water-dispersible PDMS, we first converted commercial PDMS to PDMS-COOH, as illustrated in Scheme 1. The terminal hydroxyl groups of PDMS reacted with MBTCA, and the resulting PDMS bears carboxyl functional groups (-COOH) along the polymer backbone.

**Figure 2:**
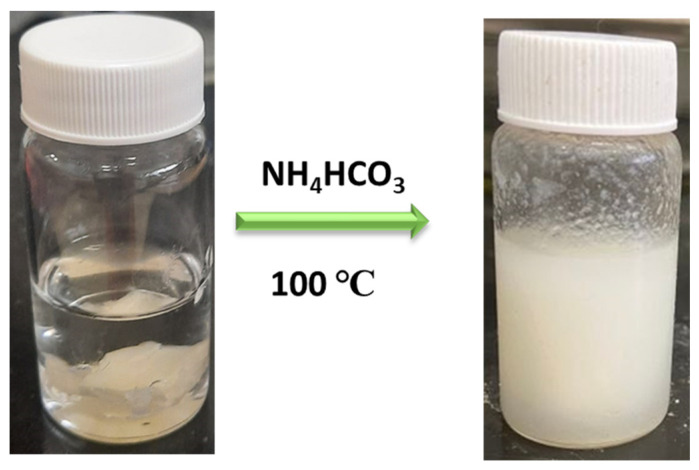
Ionization of PDMS-COOH using ammonium carbonate to obtain a water-based white emulsion that we used for paper coating.

Based on the ^1^H-NMR spectrum (Figure 1), the reaction conversion calculated from the integration of the methylene protons of DMS-C16 (-CH_2_ in red color) and the methine protons of MBTCA (-CH in yellow color) is 97%, which is compatible with the reaction yield calculated based on the resulting PDMS weight, 98%. The synthesized PDMS-COOH was ionized to make an emulsion with ammonium bicarbonate. Scheme S1 illustrates the chemistry of the emulsion formation, while the resultant white emulsion obtained is shown in Figure 2. The pressure was generated during the formation of the emulsion due to the release of carbon dioxide gas that was generated by the neutralization reaction between the carboxylic acid group and ammonium bicarbonate. Therefore, some holes were punched in the lid of the vial to release the pressure. Overall, a 100% waterborne, ionic emulsion of PDMS-COOH was successfully obtained and was ready to use in paper coating. The resultant PDMS-COOH emulsion was mixed with an aqueous starch solution to prepare the final coating solution. PDMS-COOH and starch were mixed in various weight percentage ratios, as shown in Table S1. Starch was added to the PDMS-COOH to serve two purposes. Starch is used initially as a primer as a base layer to increase oil resistance and secondly to reduce tackiness offered by a neat PDMS-COOH emulsion (P1S0) in the top layer as well as to reduce the cost of coating material.

### 4.1. Basis Weight and Thickness

Table 2 lists the thickness, basis weight, coating load (g/m^2^), and coating load by wt% of paper samples that had been coated with the emulsion as well as before coating. The uncoated, blank kraft paper (B-KP) was 181.67 ± 3.40 µm thick, while the application of a coating to the sample caused the thickness to increase to 232.22 ± 10.40 µm for sample P1S0 which was coated only with the emulsion. Meanwhile, the sample P1S4 that was coated using a mixture of the emulsion and starch showed a thickness of 239.22 ± 8.81 µm. The basis weight for B-KP was 137.5 ± 0.5 g/m^2^. Meanwhile, the basis weight increased for the samples coated with the emulsion and starch blend, with values of 193.9 ± 1.7 g/m^2^ for P1S1 and 186.6 ± 2.2 g/m^2^ for P1S4. Similarly, the coating load increased with a high content of starch in comparison to that obtained with a neat emulsion of PDMS-COOH, as the coating load for P1S0 was 36.0 ± 2.5 g/m^2^ while that for P1S1 was 56.4 ± 1.7 g/m^2^.

### 4.2. FTIR Analysis

**Figure 3:**
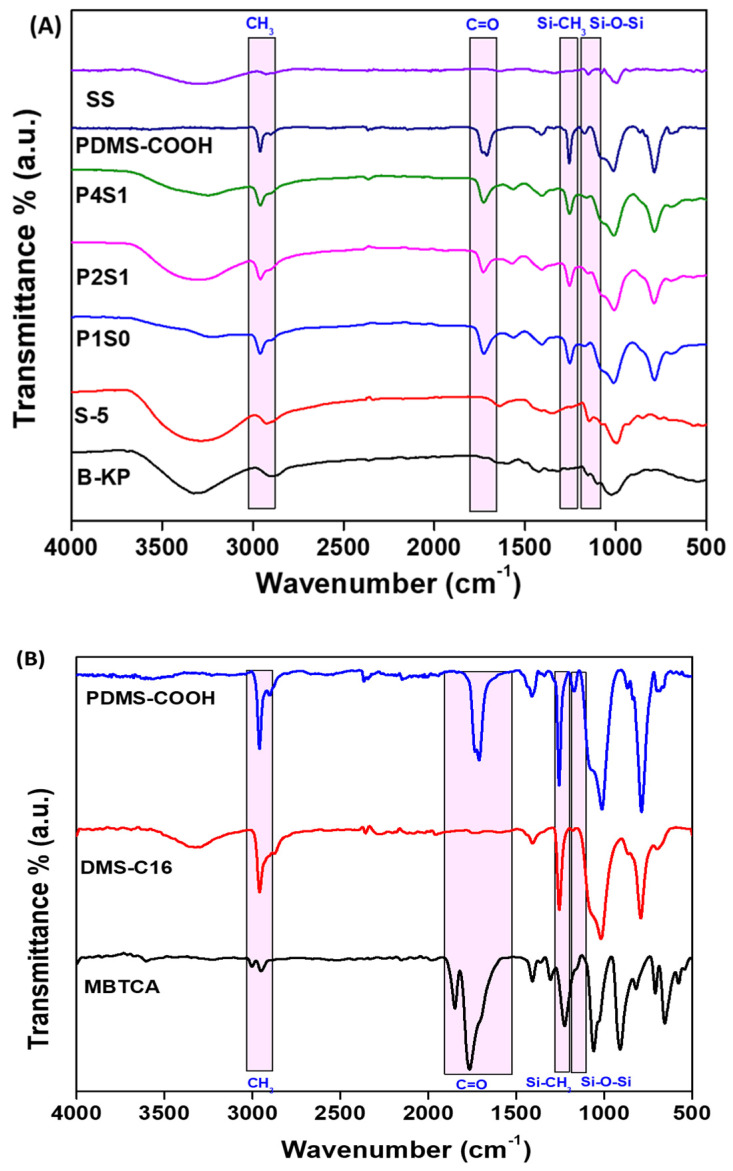
(**A**) FTIR spectra of blank kraft paper (B-KP), modified coated paper samples, and solid materials used for coating, including PDMS-CCOOH and solid starch (SS). (**B**) FTIR spectra of starting materials, *meso*-butane-1,2,3,4-tetracarboxylic dianhydride (MBTCA), carbinol (hydroxyl)-terminated polydimethylsiloxane 50–65 cSt (DMS-C16), and PDMS-functionalized carboxylates (PDMS-COOH).

ATR-FTIR spectroscopy was used to characterize the synthesized PDMS-COOH, solid starch (SS), and paper substrates used in each coating as shown in Figure 3. The two characteristic peaks corresponding to carbonyl groups in the FTIR spectrum of PDMS-COOH can be seen at 1740 and 1725 cm^−1,^ indicating the presence of ester and carboxylic acid groups, respectively [2]. The peak at 2960 cm^−1^ corresponds to a -CH_3_ asymmetric stretch and bending vibration at 1257 cm^−1^ in Si-CH_3_. In addition, the peak at 1022 cm^−1^ corresponds to Si-O-Si stretching [37]. The particular cellulose peaks appear in the specific range of 1150–1100 cm^−1^, indicating the stretching vibration of C-O, while the peak at 2900 cm^−1^ was attributed to C-H stretching vibrations. Starch-coated paper (S-5) and solid starch (SS) showed similar IR spectra to that of B-KP, as starch and cellulose have similar structures except for differences in their linkages, as cellulose has beta linkages while starch possesses alpha linkages. Therefore, both also showed a broad band around 3400, corresponding to free hydroxyl groups [51]. The peaks of PDMS-COOH can be clearly seen on coated paper samples such as P1S0, P2S1, and P4S1, showing the presence of coating material on paper surfaces, while these peaks are not observed in the FTIR spectra of B-KP, S5, and SS. In Figure 3B, the spectrum of MBTCA clearly indicates the presence of two axial deformation bands at 1844 and 1766 cm^−1^, which is characteristic of an anhydride carbonyl group [52]. These bands disappear in the FT-IR spectrum of PDMS-COOH. Instead, these bands are substituted by the two new characteristic bands at 1740 and 1725 cm^−1^, corresponding to esters and carboxylic acid carbonyls, thus indicating the conversion of anhydride groups to esters and acid functionalities. Moreover, the appearance of peaks at 2960 and at 1022 cm^−1^ which are characteristic of PDMS (DMS-C16) in the spectrum of PDMS-COOH also supports the synthesis.

### 4.3. Scanning Electron Microscopy (SEM) Analysis

**Figure 4:**
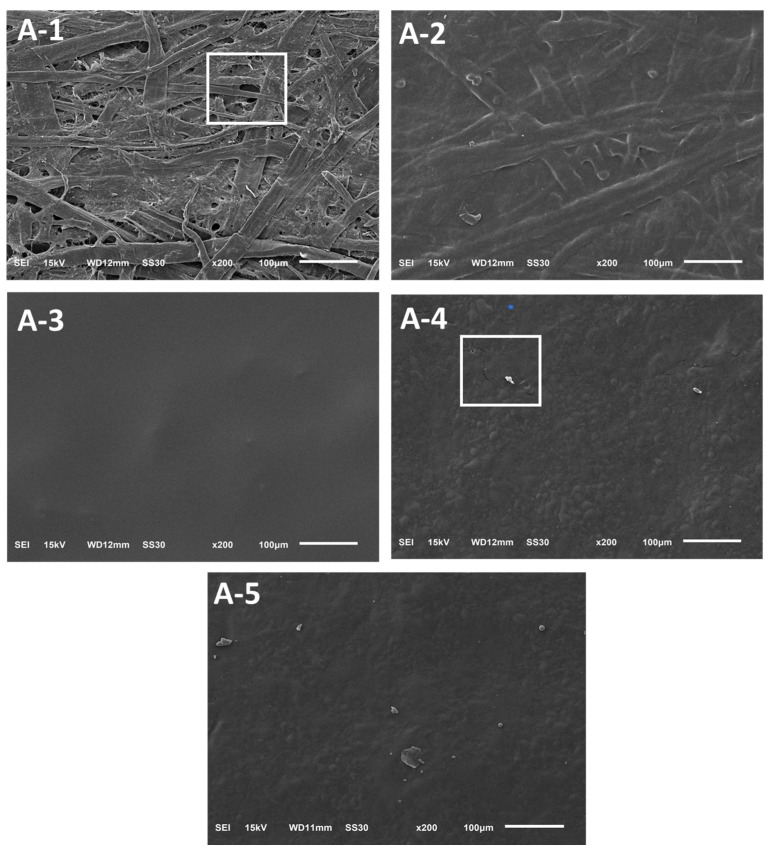

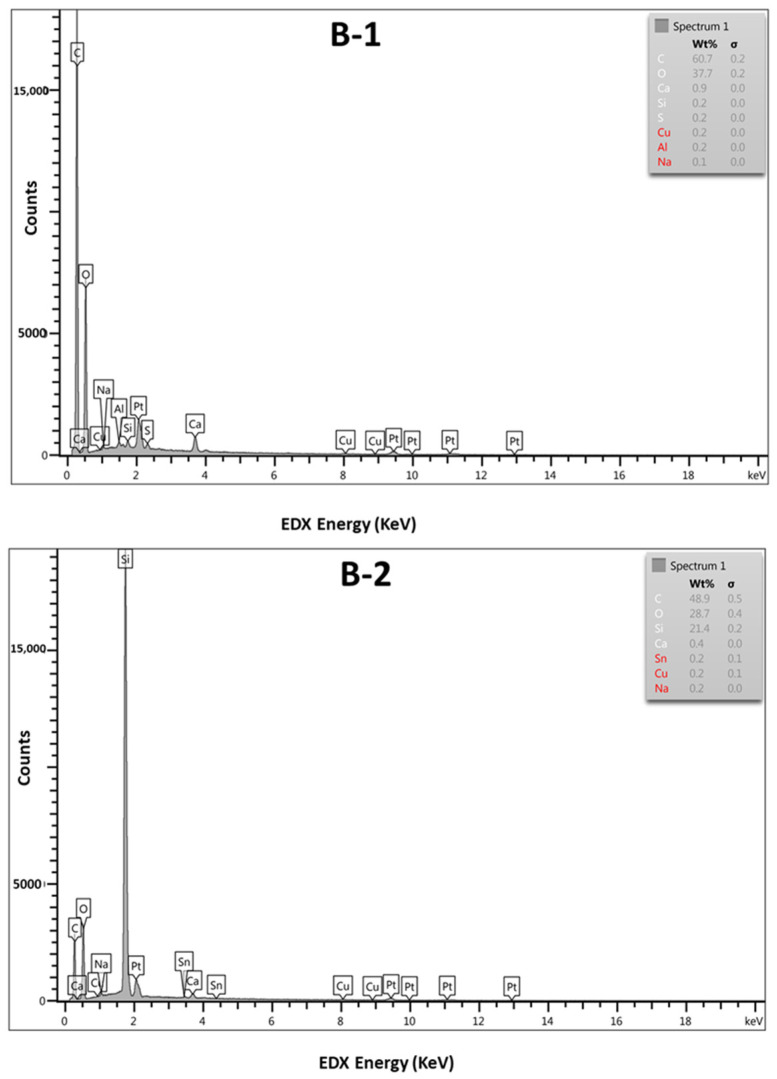
SEM images (200×) of (**A-1**) blank kraft paper (B-KP), (**A-2**) 5% starch-coated paper 5-S, (**A-3**) P1S0, (**A-4**) P2S1, and (**A-5**) P4S1. The bar scale shows 100 µm, The area in white boxes shows the pores and cracks present on paper surface. EDX spectra of blank kraft paper (**B-1**) and P4S1 (**B-2**).

**Figure 5:**
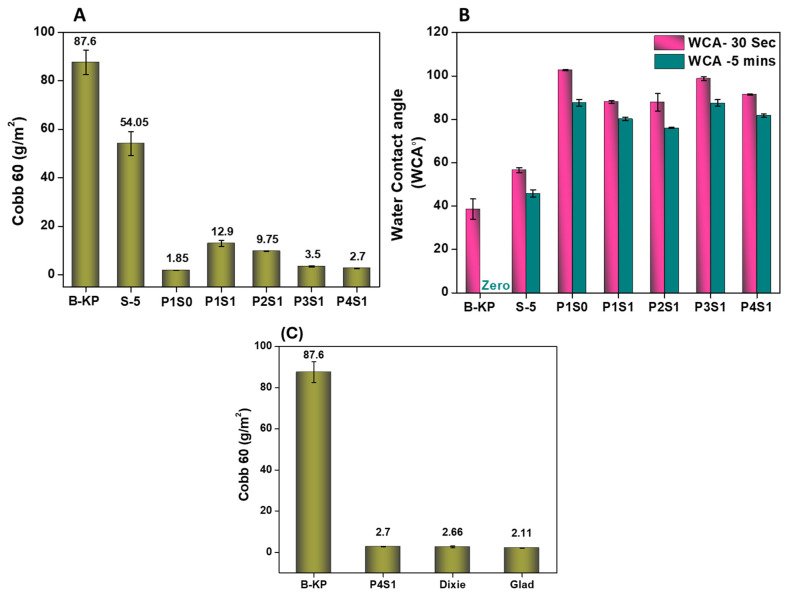
(**A**) Cobb60 values (g/m^2^) of blank kraft paper and coated paper samples using various concentrations of PDMS-COOH and starch. (**B**) Water contact angles (WCAs) measured after the intervals of 30 and 5 min of putting water droplets on the surface of blank kraft paper and various coated paper samples. (**C**) A comparison of the Cobb60 values (g/m^2^) of blank kraft paper, coated paper P4S1, and two commercial control paper plates.

The SEM analysis showed that all the pores were nicely masked for coated paper, which resulted in an improvement in water and oil resistance, enhanced barrier properties, and a reduction in the surface energy of the coated samples. A valuable insight in this regard was obtained via SEM analysis (Figure 4). The images recorded at 200× clearly demonstrate that there were holes and channels in the unmodified paper (B-KP, Figure 4(A-1)). The pores in kraft paper are fully covered by the starch base layer coating, owing to the excellent film-forming ability of starch. It should be noted that SEM imaging reveals the underlying roughness of the paper because the starch layer was not thick enough to cover all the roughness. A clear, smooth surface was obtained with the coating of the PDMS-COOH emulsion, as can be seen in the SEM photographs of P1S0 (Figure 4(A-3)). When there was a high content of starch, some cracks can be seen in the surface, as was the case with P2S1 (Figure 4(A-4)). In contrast, the use of a small ratio of starch with emulsion, as was the case with P4S1 (Figure 4(A-5)), yielded a smooth coating surface that covered all pores without exhibiting visible cracks. In Figure 4(A-3,A-5), the roughness disappears due to the increased layer thickness. Figure 4(A-4,A-5) also show evidence of phase segregation between starch and PDMS-COOH. Figure 4(B-1,B-2) show a comparison of the elemental percentages in blank uncoated paper (Figure 4(B-1)), where 60.7% carbon (C) and 37.7% oxygen (O) were detected, while silicon (Si) was only found at trace levels (i.e., 0.2%). The incorporation of coating material onto paper sample P4S1 increased the Si content to 21.4%, while the C and O contents were 48.9% and 28.7%, respectively.

### 4.4. Water Resistance

The main concern with paper-based packaging material is the absorbance of moisture during storage, which leads to poor mechanical strength. The reason for this deterioration in mechanical strength is due to the porous nature of paper. This problem can be solved by using a hydrophobic coating material that can not only cover the pores in the surface paper substrate but also improve the water repellency of paper-based packaging materials and thus improve their barrier properties against water vapors.

### 4.5. Liquid Water Resistance

The water resistance of the paper sample was explored by recording the Cobb60 values (Figure 5A). The uncoated paper immediately absorbed a lot of water and showed a very high Cobb60 value of 87.6 ± 5.1 g/m^2^ due to its highly porous structure and water uptake on the surfaces of the cellulose fibers. The application of a starch layer caused the pores of the paper substrate to become covered, thus reducing the Cobb60 value to 54.05 ± 4.87 g/m^2^. As starch is a very hydrophilic material, only a modest reduction in the Cobb60 value was observed, although the pores of the paper were nicely masked. In contrast, the application of the PDMS-COOH emulsion resulted in a dramatic reduction in the Cobb60 value to 1.85 ± 0.07 g/m^2^. There was a slight rise in the Cobb60 values when the emulsion was mixed with starch (particularly with higher ratios of starch as was the case with P1S1) up to 12.90 ± 1.27 g/m^2^ due to the hydrophilic nature of starch [4]. On the other hand, the sample with a small ratio of starch (P45S1) showed a very low Cobb60 value of 2.7 ± 0.14 g/m^2^, which was comparable to that exhibited by the paper that had been coated only with the neat PDMS-COOH emulsion. The water resistance of coated paper samples was increased in comparison to our previous reported work [1] where we used chitosan-*grafted* PDMS (CHI–*g*–PDMS) as a coating and found that the Cobb60 values were reduced by up to 50% in comparison with uncoated paper. In this study, there were 85–97% reductions in the overall Cobb60 values of the coated paper samples in comparison with those of uncoated paper, rendering them highly water-repellent.

Figure 5B depicts the water contact angle (WCA) values, which were measured to further support the water resistance of modified paper substrates against liquid water. The droplet was applied, and WCAs were recorded after the intervals of 30 s and 5 min, and results observed with coated paper samples were compared with those observed with non-coated paper. The result showed that B-KP initially exhibited a WCA of 38.55 ± 4.73°. The water was quickly taken up by the paper, with a dark stain being left behind after 5 min with a WCA of zero. The WCA for P1S0 increased significantly to 102.65 ± 0.21° at 30 s, and even after passing 5 min, it was higher than that observed on uncoated paper (i.e., 87.65 ± 0.61°), thus indicating that the coated paper sample P1S0 had increased water repellency. Similar results were noted for sample P1S4, with a WCA of 91.40 ± 0.28°, which was reduced to 81.80 ± 0.70° at 5 min after placing the droplet. The WCAs showed that the water repellency of coated papers was increased with coating, and these results were consistent with the Cobb60 values. In general, the increase in WCA for coated paper samples showed the coated paper samples are water-repellent.

To evaluate the practical applicability of our designed coated papers as water-resistant paper, the results obtained with uncoated paper and the selected best-performing sample P4S1 (with low Cobb60 values and no stickiness) were compared with commercial controls, namely Dixie (acrylic-based coated plate) and Glad (low-density polyethylene-coated paper) paper plates (Figure 5C). It was observed that the Cobb60 values of our designed coated paper sample P4S1 were comparable and close to the Cobb60 values offered by commercial benchmarks, suggesting that it could be a promising candidate for commercial applications as a water-resistant paper product.

To see the effect of liquid water on coated paper surfaces, the droplet test was performed by keeping a water droplet on the surface (Figure S1). The images were captured after 30 s and 5 min and, finally, after the droplet had been wiped off the surface using a chem wipe to explore whether any traces of the droplet remained. The images show that water has been taken up by the B-KP quickly, which is shown by the appearance of a dark brown spot on the paper after the application of the water droplet that indicated the water absorption. Similarly, a light spot appeared on the S-5 paper. In contrast, there were no stains or spots observed on the surfaces of the P1S0 and P1S4 samples, suggesting that they were water-repellent.

### 4.6. Water Vapor Barrier Properties

To evaluate the barrier properties of our coated paper samples, the water vapor transmission rate (WVTR) was measured at 23 °C and 50% RH, as shown in Figure S2. The uncoated paper, B-KP, due to its porous structure, showed a high WVTR value of 1128.41 ± 169.76 g/(m^2^-day). Interestingly, the paper substrates coated with starch 5% (S-5) have also shown a decrease down to 384.68 ± 21.62 g/(m^2^-day) because all the pores are nicely masked by the starch layer, which reduced the passage of water vapors. The application of the PDMS-COOH coating further reduced the WVTR value. Sample P1S0 showed a WVTR value of 246.47 ± 18.98 g/(m^2^-day), and sample P4S1 showed a WVTR value of 308.90 ± 7.63 g/(m^2^-day). In general, there was around a 75% reduction in WVTR, which showed the coated paper possesses high water barrier properties.

### 4.7. Oil Resistance

**Figure 6:**
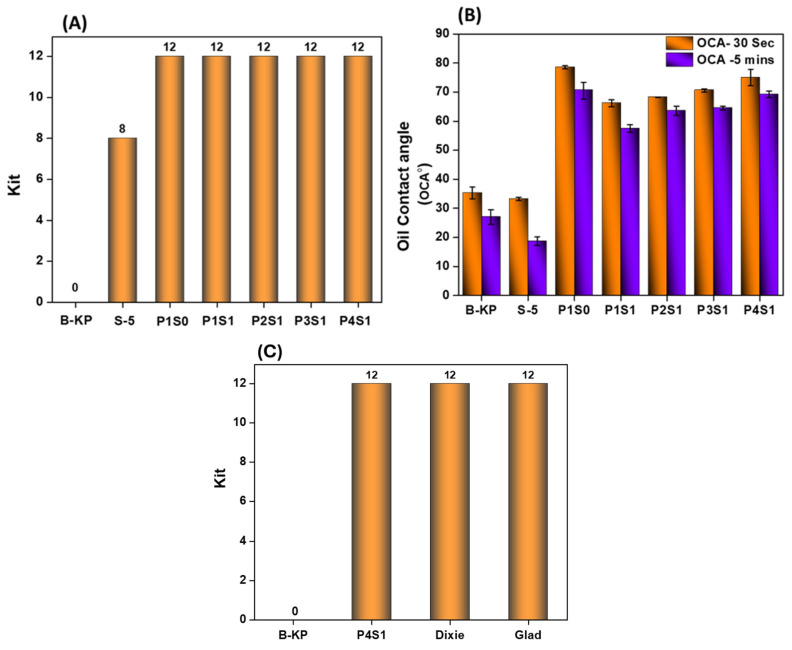
(**A**) Kit ratings of blank kraft paper and paper samples coated using various concentrations of PDMS-COOH and starch. (**B**) Oil contact angles (OCAs) were measured after the interval of 30 and 5 min of putting castor oil droplets on the surface of blank kraft paper and various coated paper samples. (**C**) A comparison of the kit ratings of blank kraft paper, coated paper P4S1, and two commercial control paper plates (Dixie and Glad).

Figure 6A shows the kit test values of paper samples, showing the oil resistance of coated paper samples in contrast to B-KP, indicating that oil resistance has improved from 0 (failure of kit number 1 is considered as zero) to kit number 12. The kit numbers are assigned based on the kit solutions passed by a sample (leaving no darkness on application). These solutions are numbered from 1–12, which vary in ratios of castor oil, toluene, and *n*-heptane. The higher the kit number possessed by a sample, the better the oil resistance associated with it. The result revealed that B-KP absorbed oil quickly and showed a kit rating of 0 (as it failed the test performed by the liquid with the lowest kit number of 1), while the application of starch (to obtain S-5) caused the kit rating to increase to 8. The application of the PDMS-COOH coating layer further enhanced the oil resistance up to a kit number of 12, which corresponds to the highest oil resistance on this scale. Compared to our previously reported coated paper samples [1] that were prepared using CHI-*g*-PDMS and which exhibited a kit rating of 11, this coated paper showed an increase in its kit rating up to 12. The result shows that PDMS-COOH-coated paper possesses excellent oil resistance.

The oil resistance of the coated paper samples was further explored by determining their oil contact angles (OCAs) as shown in Figure 6B. These tests were performed in a similar manner to the WCA measurements, using castor oil droplets. The result revealed that the initial B-KP sample showed an OCA of 35.30 ± 1.97° at 30 s, which was reduced to 27.00 ± 2.54° after 5 min had elapsed, leaving a very dark stain on the paper, thus indicating the absorbance of oil by the B-KP paper sample. There was a significant increase in the OCA associated with coated paper up to 78.50 ± 0.56° for P1S0 in 30 s. There was a very small reduction in the OCA after a 5 min time interval (i.e., 70.55 ± 2.89°), demonstrating that the coated paper had high oil repellency. It was noted that the incorporation of starch led to a small decrease in the OCA because the resultant coating exhibited a highly smooth surface. Contact angles are dependent on the smoothness of the surface [23], and the droplet was spread on paper but did not penetrate the paper, as demonstrated by the absence of dark stains during testing.

To establish the practical applicability of our proposed coated papers as oil-resistant products, the kit ratings of uncoated paper and sample P4S1 were also compared with those of commercial controls (i.e., Dixie and Glad paper plates). It was found that the kit rating of sample P4S1 is equal to those of commercially available Dixie and Glad plates. This impressive performance shows that our designed coated paper can be used as an oil-resistant paper product.

The behavior of castor oil droplets kept on the surface of paper was explored in the same manner as the water droplet test, using castor oil (Figure S3). The results revealed that the oil droplet was quickly absorbed by B-KP, while in comparison to coated paper samples, it presented no sign of oil droplet absorption. Once the oil droplet was removed, the paper looked exactly like it had prior to the placement of the oil droplets, which is consistent with the kit rating data and suggests that the coated paper samples have good oil resistance.

### 4.8. TGA Analysis

The thermal stability of coated paper samples was established using TGA analysis in comparison to solid material used in coating and blank kraft paper. The results obtained are presented in TGA and DTG curves (Figure S4). In the plot, the weight loss observed below 110 °C is attributed to the evaporation of moisture (i.e., the water absorbed by the surface of cellulose fibers). The result demonstrates that B-KP decomposed at ~380 °C. The solid PDMS-COOH had two decomposition points, at ~350 °C and ~450 °C. Meanwhile, the coated paper samples underwent decomposition at around ~390 °C, and their peaks almost overlapped that of B-KP due to their small coating content, thus suggesting that they did not adversely affect the thermal stability of the paper substrate. The decomposition of coated paper above 350 °C [18] renders them a good candidate for use as packaging materials where hot meals are packaged and presented. As the coated paper decomposes at high temperatures (such as 390 °C), it can find its application in food packaging for high-temperature needs.

### 4.9. Mechanical Properties

**Figure 7:**
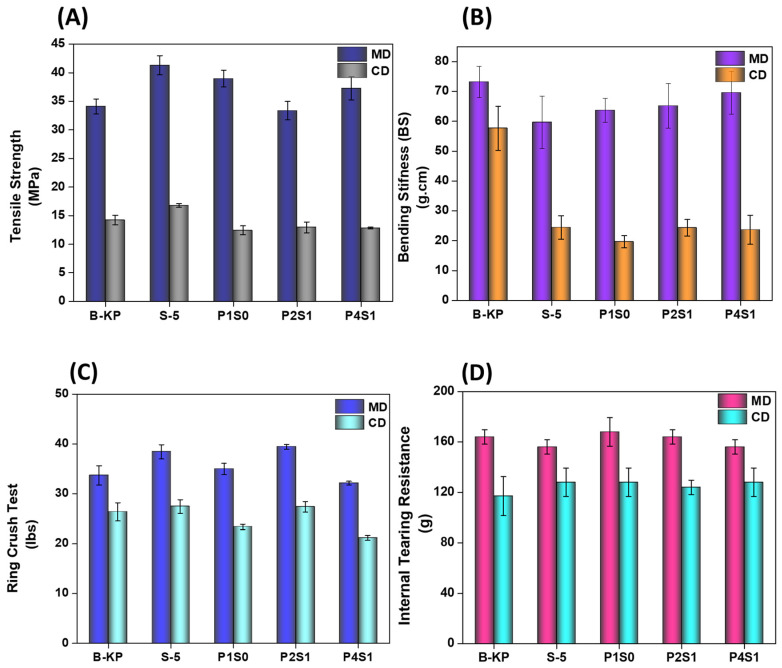
Tensile strength (**A**), bending stiffness (**B**), ring crush test (**C**), and internal tearing resistance (**D**) of blank kraft paper and coated paper samples.

The integrity of a package is dependent on its mechanical properties; therefore, they play a crucial role in the design of packaging supplies. To analyze the mechanical properties of our coated paper samples, a series of tests were conducted, as shown in Figure 7A–D. The tensile strength (Figure 7A) was recorded and corresponded to the maximum strength a material can withstand when stretched. The uncoated paper sample B-KP had a tensile strength of 34.1 ± 1.32 MPa, which increased slightly after the application of the coating material to 37.26 ± 2.05 MPa in the MD for sample P4S1. The results for the CD showed that the sample maintained its tensile strength after the application of the coating. Bending stiffness (BS) is the property of a sample that shows its resistance against bending deformation. The BS value values were recorded and are presented in Figure 7B. The BS value of uncoated paper is ~73.16 ± 5.25 g·cm in the MD. The coated sample P4S1 retained the BS value in the MD. The BS value in the CD was 57.66 ± 7.37 g·cm for B-KP, which was reduced to 23.66 ± 4.85 g·cm for P4S1, showing that the presence of the coating caused the bending strength to be reduced in the CD due to possible elimination of intra-cellulose hydrogen bonding. The RCT values were measured and are shown in Figure 7C. B-KP exhibited an RCT value of 33.70 ± 1.93 lbs in the MD and 26.36 ± 1.80 lbs in the CD. For the coated paper sample P4S1, the RCT value was found to be 32.13 ± 0.37 lbs in the MD and 21.16 ± 0.49 lbs in the CD. The represented coated paper sample P4S1 has retained its RCT by up to 95% in the MD and 80% in the CD. Similar retention of mechanical properties was observed with the ITR (Figure 7D), as the coated samples showed slight variation in properties that were within standard deviation limits.

The remaining mechanical properties, including bursting tests, % elongation at break, and Young’s modulus, are also measured and are presented in Figure S5. The overall results from all tests carried out to study mechanical properties showed there was 70–95% retention of these mechanical properties in comparison with B-KP after coating, thus suggesting that the coated paper can provide an excellent packaging material while maintaining its integrity.

### 4.10. Recyclability

**Figure 8:**
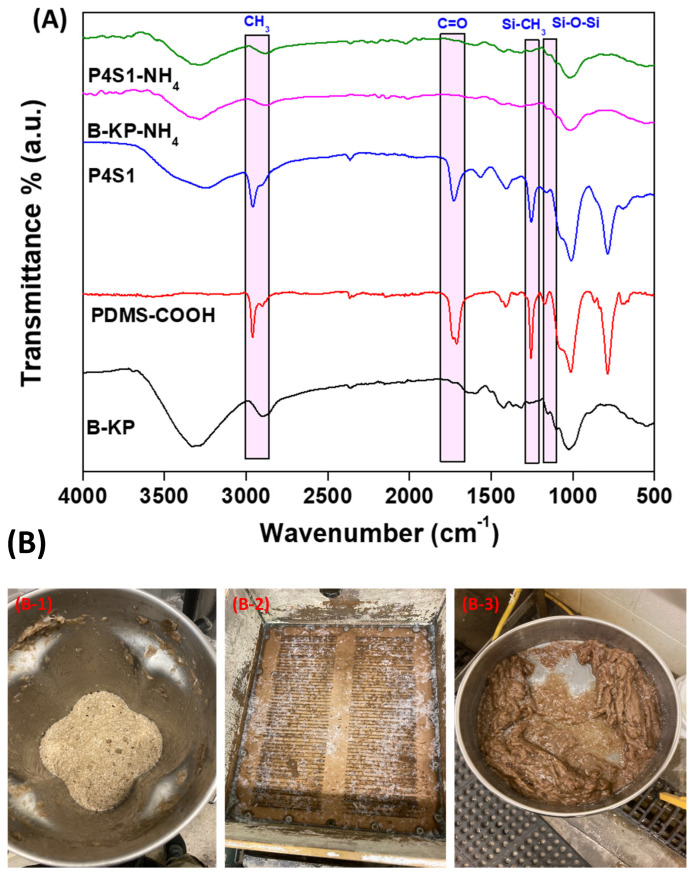
(**A**) FTIR spectra of recycled paper samples, which included blank kraft paper (B-KP), recycled B-KP that had been washed with NH_4_HCO_3_ (B-KP-NH_4_), recycled paper P4S1 that had been washed with NH_4_HCO_3_, and PDMS-COOH solid material. (**B**) The sample P1S0 after repulping (**B-1**), screen-rejected material of P1S0-coated paper (**B-2**), and screen-accepted material of P1S0 (**B-3**).

Pulp recovery is a very important aspect of paper packaging as it trims down the burden on landfill management. The sample P4S1 was selected for repulping as it contains both PDMS-COOH and starch content. It was recycled via the process depicted in Scheme S2. The sample B-KP was used as a control to see the effect of ammonium bicarbonate washing on the pulp itself. ATR-FTIR analysis of recycled paper (P4S1-NH4) was performed to confirm the separation of the coating material from the pulp. The disappearance of peaks corresponding to PDMS-COOH in the regions of 2960, 1740, 1725, 1257, and 1022 cm^−1^ in the FTIR spectra of P4S1-NH4 confirmed this separation. The ATR-FTIR (Figure 8A) spectrum was recorded for the sample B-KP that had been washed with ammonium bicarbonate and is labeled as B-KP-NH4. Thus, the coated paper can be recycled via the pulp recovery method, thus promoting the circular economy.

Figure 8(B-1–B-3) show the general appearance of repulping and rejects during the test. The percentage yield of the repulping process was 91.1%, which shows that coated paper sample P1S0 has passed the test. The overall results are shown in Table 3.

## 5. Conclusions

In summary, we have developed a greener (solvent-free) method for the fabrication of water- and oil-resistant coated paper using a PDMS-COOH emulsion. The PDMS-COOH was synthesized in solvent-free conditions, and its successful synthesis was confirmed by FTIR and ^1^H-NMR spectroscopy techniques. The synthesized PDMS-COOH was emulsified using ammonium bicarbonate in water. The emulsion was then blended with a starch solution in various concentrations and then coated onto kraft paper, which had been precoated with a 5% starch solution. The ammonia and moisture from the coated paper sample were removed by drying the coated paper in an oven at 130 °C. All coated papers were characterized via FTIR and SEM analyses. A very good water and oil resistance has been presented by coated paper substrates using various amounts of PDMS-COOH and starch with up to 97% enhancement in water resistance being shown by the best-performing sample (P4S1) in the tested series prepared by blending with starch, as compared to blank kraft paper and kit ratings up to 12 (highest m number on kit rating scale). The water and oil resistance of sample P4S1 was comparable to that of commercial controls, suggesting that it could be a potential candidate for commercial application. The coated paper substrates showed a substantial enhancement in water barrier properties tested at 50% RH and 23 °C. TGA analysis supported the practical applicability of coated papers at high temperatures. In addition, the paper was successfully recycled via pulp recovery with a percentage yield of 91.1%. Considering the growing environmental concerns due to the high content of fluorine-containing compounds, this study can offer a potential alternative to single-use plastics. Initial cost analysis suggests that the coating material has a modest price of USD 5.45. We are currently evaluating the cost-effectiveness of producing paper coated with water-dispersible poly(dimethylsiloxane) in comparison to PFAS-coated paper and single-use plastics.

## Figures and Tables

**Table 1:** Selected formulations and sample codes used in this study.

Abbreviated Name	PDMS-COOH %	Starch %
B-KP ^a^	-	-
S-5 ^b^	-	-
P1S0 ^c^	100	0
P1S1 ^d^	50	50
P2S1 ^e^	66.6	33.3
P3S1 ^f^	75	25
P4S1 ^g^	80	20

^a^ Kraft paper (B-KP); ^b^ paper coated with 5% starch as a base layer; ^c^ coated paper obtained using 100% emulsion only; ^d^ coated paper with 50:50% of emulsion and starch; ^e^ coated paper prepared using 66.6% emulsion and 33.3% starch; ^f^ coated paper prepared using 75% emulsion and 25% starch; ^g^ coated paper prepared using 80% emulsion and 20% starch.

**Table 2:** Thickness, basis weight, and coating load of uncoated and coated paper samples.

Sample No.	Material Thickness (µm)	Basis Weight (g/m^2^)	Coating Loading (g/m^2^)	Coating (Load by wt%)
B-KP	181.7 ± 3.4	137.5 ± 0.5	0	0
S-5	201.0 ± 5.6	145.2 ± 0.6	7.7 ± 0.7	5.36 ± 0.43
P1S0	232.2 ± 10.4	173.5 ± 2.5	36.0 ± 2.5	26.99 ± 2.90
P1S1	261.5 ± 15.1	193.9 ± 1.7	56.4 ± 1.7	41.43 ± 0.51
P2S1	232.3 ± 5.9	185.5 ± 0.6	48.0 ± 0.5	34.82 ± 0.43
P3S1	278.1 ± 6.9	183.5 ± 1.1	46.0 ± 1.1	32.72 ± 0.36
P4S1	239.2 ± 8.8	186.6 ± 2.2	49.0 ± 2.3	35.41 ± 1.59

**Table 3:** Percentage yield of the repulping process for sample P1S0.

Content	Unit	P1S0
Moisture	(wt%)	6.12
Sample charged	(g)	25.00
Screen reject	(g)	1.83
Screen accepts	(g)	18.79
Yield of sample	(wt%)	91.10
Pass/Fail		Pass

## Data Availability

Data are contained within the article and Supplementary Materials.

## Supplementary Materials

The following supporting information can be downloaded at: https://www.mdpi.com/article/10.3390/polym16071006/s1, Scheme S1: Ionization of PDMS-COOH using ammonium bicarbonate in water; Scheme S2: Paper recycling via the repulping approach using blank kraft paper and P1S4-coated paper; Figure S1: Photographs of blank kraft paper (B-K) and coated paper samples (S-5, P1S0, and P4S1) before the application of water droplets, 5 min after the application of water droplets, and after the removal of the water droplets; Figure S2: WVTR (g/m^2^-day) of blank kraft paper and coated paper samples at different %RH (50% & 90%) and temperatures (23 & 38 °C); Figure S3: Photographs of uncoated (B-KP) and coated paper samples (S-5, P1S0, and P4S1) before the application of castor oil droplets, 5 min after the application of these droplets, and after the removal of these droplets; Figure S4: TGA plots of blank kraft paper, coated paper samples, and samples of solid coating material. (B) DTG plots of blank kraft paper, coated-paper samples, and solid material used in coating including PDMS-COOH and solid starch (SS); Figure S5: % Elongation at break (A), Young’s modulus (B), and bursting test (C) of blank kraft paper and coated paper samples; Figure S6: Effect of treatments on water contact angle after 5 min; Figure S7: Effect of treatments on oil contact angle after 5 min; Figure S8: Effect of treatments on tensile strength (MD); Figure S9: Effect of treatments on tensile strength (CD); Figure S10: Effect of treatments on % elongation (MD); Figure S11: Effect of treatments on % elongation (CD); Figure S12: Effect of treatments on Young’s modulus (MD); Figure S13: Effect of treatments on Young’s modulus (CD); Table S1: Selected formulations and corresponding codes used in this study; Table S2: Tukey multiple mean comparisons of treatment effects on water contact angle after 5 min; Table S3: Tukey multiple mean comparisons of treatment effects on oil contact angle after 5 min; Table S4: Tukey multiple mean comparisons of treatment effects on tensile strength (MD); Table S5: Tukey multiple mean comparisons of treatment effects on tensile strength (CD); Table S6: Tukey multiple mean comparisons of treatment effects on % elongation (MD); Table S7: Tukey multiple mean comparisons of treatment effects on % elongation (CD); Table S8: Tukey multiple mean comparisons of treatment effects on Young’s modulus (MD); Table S9: Tukey multiple mean comparisons of treatment effects on Young’s modulus (CD); Table S10: Summary of literature on paper coating approaches showing water and oil resistance coating; References [1,23,38,39] have been cited in the Supplementary File.
