# Supplementary material for: Synthesis of Water-Dispersible Poly(dimethylsiloxane) and Its Potential Application in the Paper Coating Industry as an Alternative for PFAS-Coated Paper and Single-Use Plastics
Source: Polymers (Basel). 2024 Apr 7;16(7):1006. doi: 10.3390/polym16071006 (PMC11014279; doi:10.3390/polym16071006)
Supplement: Supplementary file 1 [file polymers-16-01006-s001.zip › polymers-2921145-supplementary.pdf]

polymers

Supplementary Materials

Synthesis of Water-Dispersible Poly(dimethylsiloxane) and Its Potential Application in the Paper Coating Industry as an Alternative for PFAS-Coated Paper and Single-Use Plastics

Syeda Shamila Hamdani [1,†], Hazem M. Elkholy [1,†,‡], Alexandra Alford [1], Kang Jackson [1], Muhammad Naveed [1], Ian Wyman [1], Yun Wang [2], Kecheng Li [2], Syed W. Haider [3] and Muhammad Rabnawaz [1,*]

[1] School of Packaging, Michigan State University, 448 Wilson Road, East Lansing, MI 48824, USA; hamdanis@msu.edu (S.S.H.); elkholyh@msu.edu (H.M.E.)
[2] Department of Chemical and Paper Engineering, Western Michigan University, 1903 W, Michigan Avenue, Kalamazoo, MI 49008, USA
[3] Department of Civil & Environmental Engineering, Michigan State University, East Lansing, MI 48824, USA
[*] Correspondence: rabnawaz@msu.edu
[†] These authors contributed equally to this work.
[‡] H.M.E. is on leave from the Department of Chemistry, Faculty of Science, Tanta University, Tanta 31527, Egypt.

Table S1. Selected formulations and corresponding codes used in this study.

Abbreviated name	PDMS (g)	Starch (g)	NH_4HCO_3 (g)
B-KP	-	-	-
S-5	-	-	-
P1S0	1	0	0.20
P1S1	0.5	0.5	0.10
P2S1	0.66	0.33	0.14
P3S1	0.75	0.25	0.17
P4S1	0.8	0.2	0.186

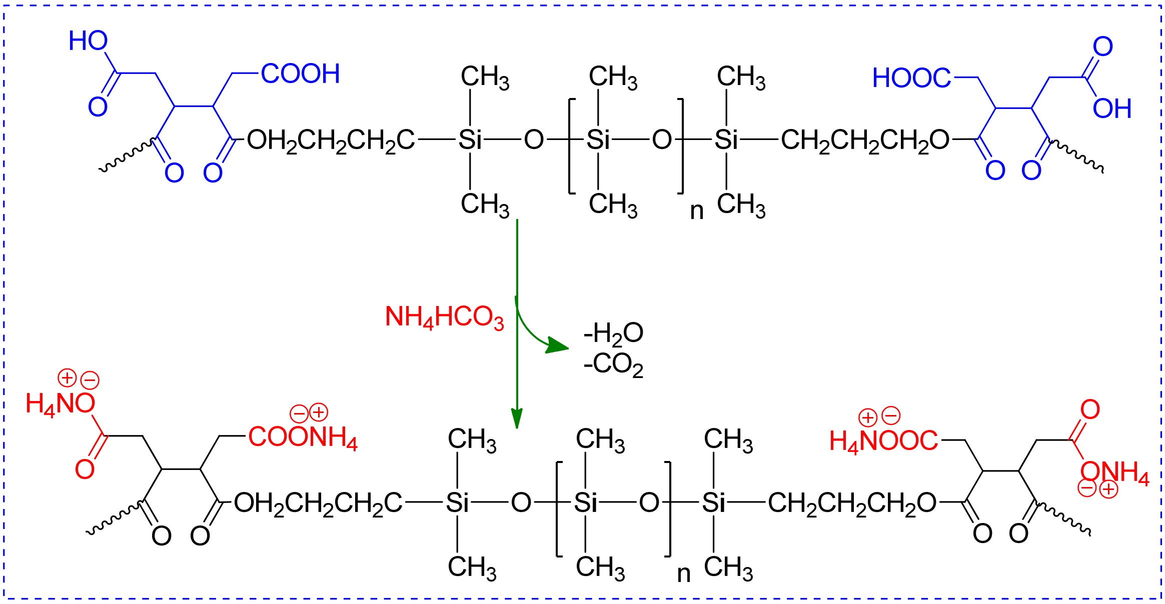

Scheme S1. Ionization of PDMS-COOH using ammonium bicarbonate in water.

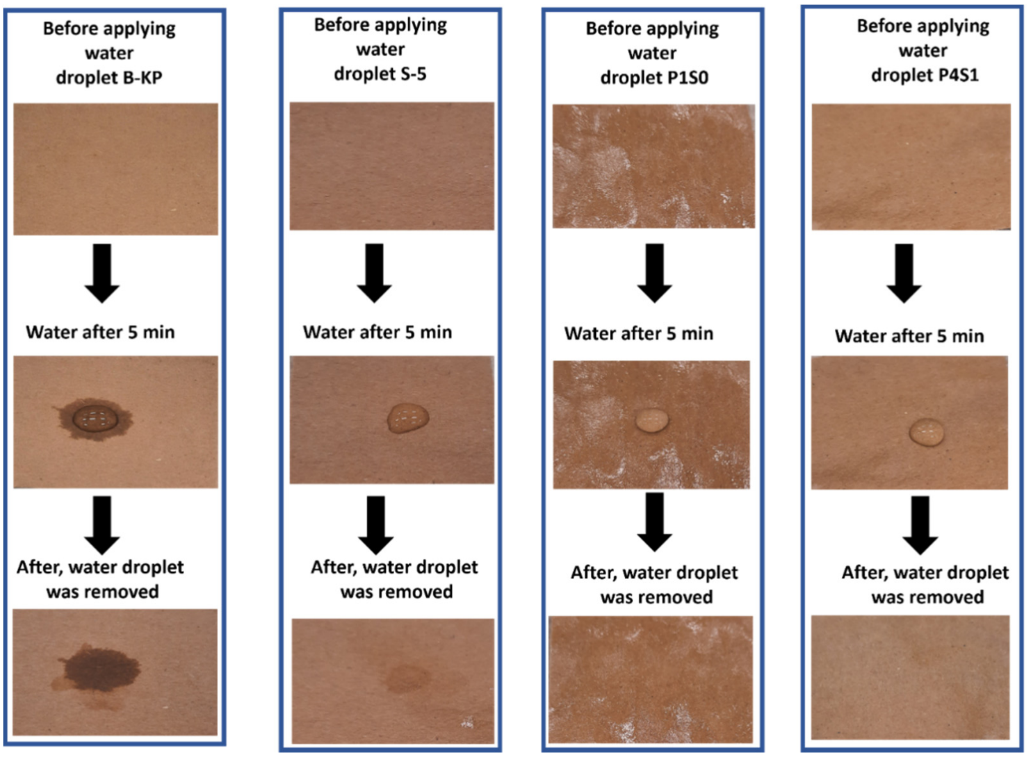

Figure S1. Photographs of blank kraft paper (B-K) and coated paper samples (S-5, P1S0, and P4S1) before the application of water droplets, 5 min after the application of water droplets, and after the removal of the water droplets.

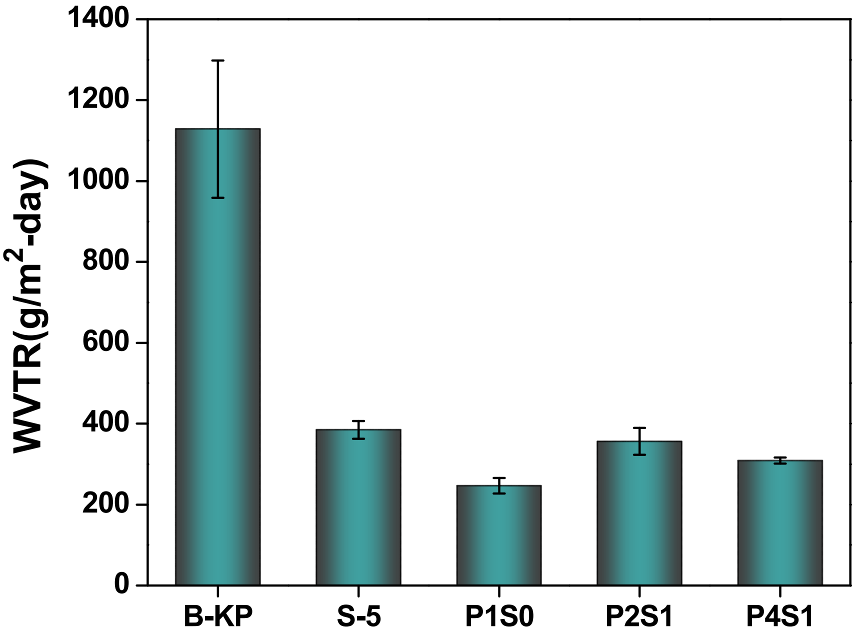

Figure S2. WVTR (g/m^2-day) of blank kraft paper and coated paper samples at different %RH (50% & 90%) and temperatures (23 & 38 °C).

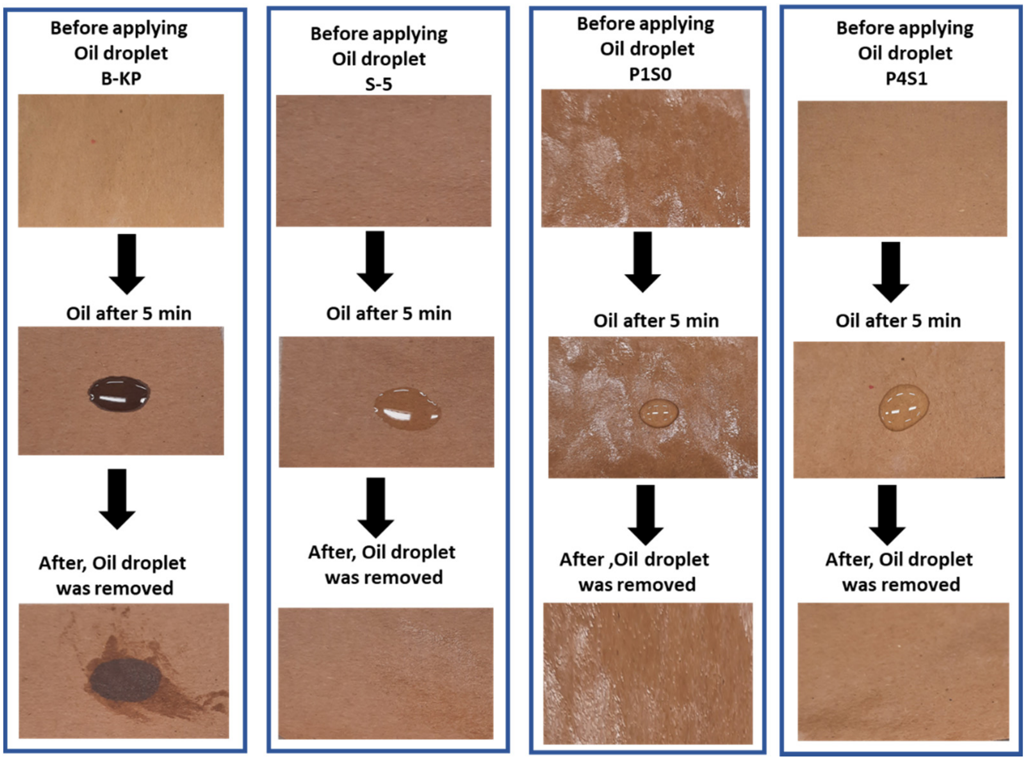

Figure S3. Photographs of uncoated (B-KP) and coated paper samples (S-5, P1S0, and P4S1) before the application of castor oil droplets, 5 min after the application of these droplets, and after the removal of these droplets.

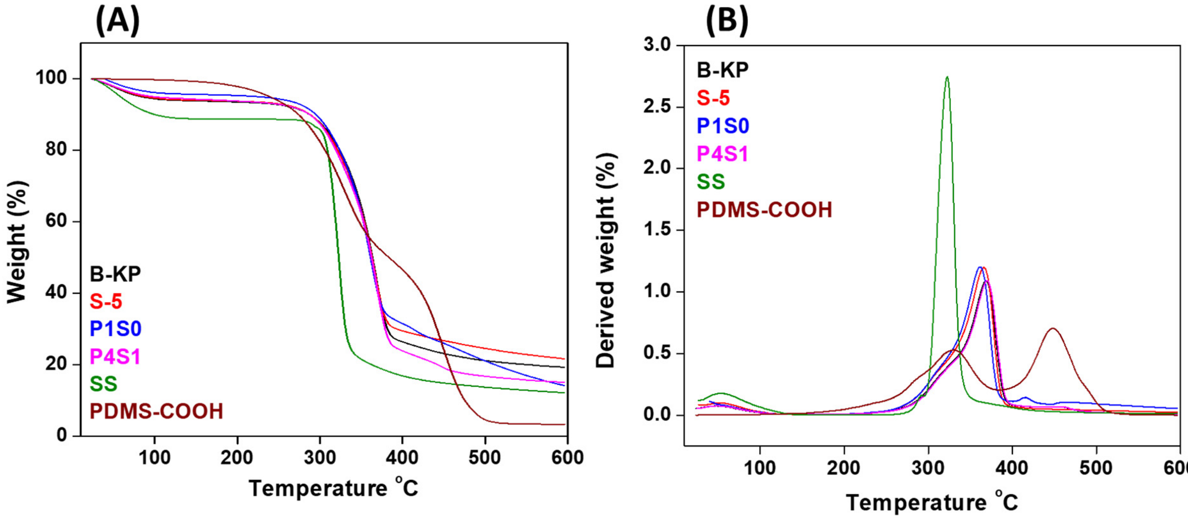

Figure S4. (A) TGA plots of blank kraft paper, coated paper samples, and samples of solid coating material. **(B)** DTG plots of blank kraft paper, coated-paper samples, and solid material used in coating including PDMS-COOH and solid starch (SS).

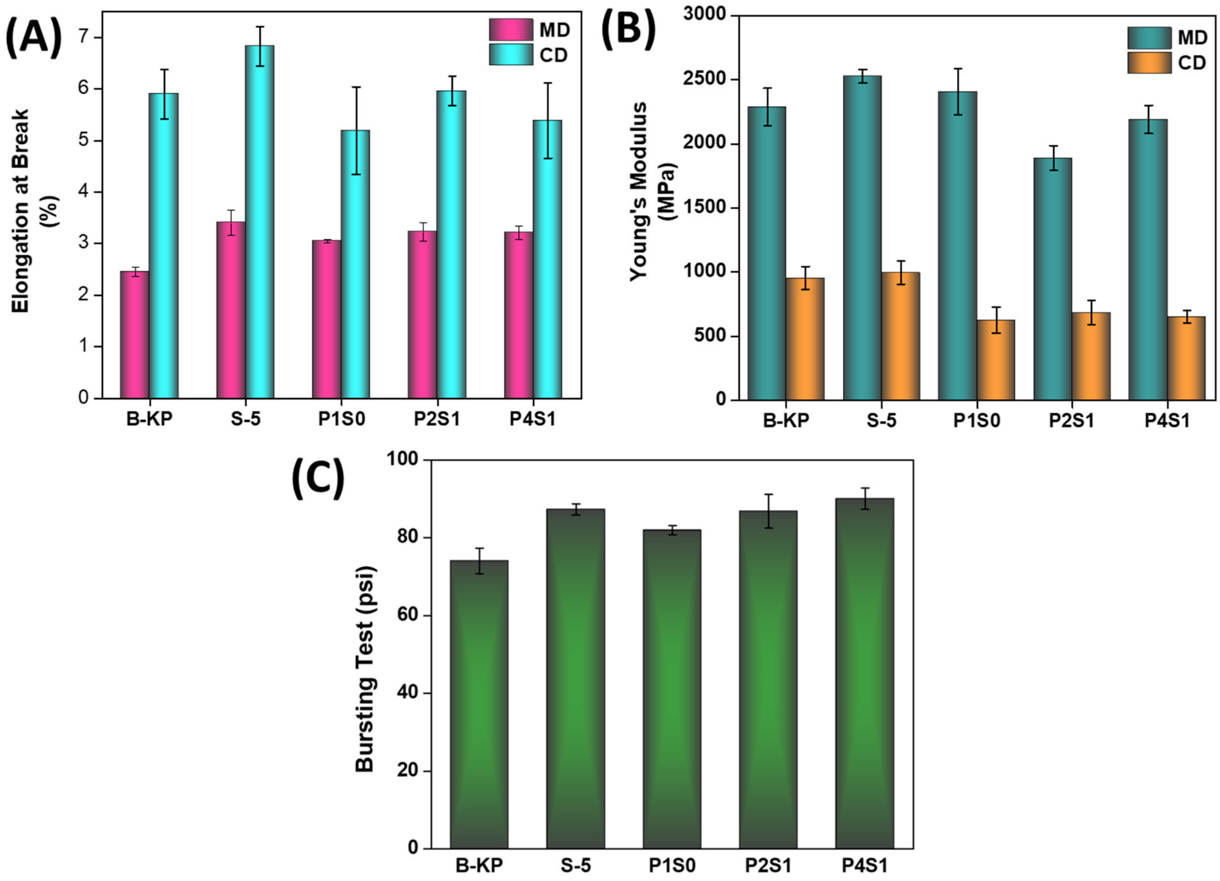

Figure S5. % Elongation at break (A), Young's modulus (B), and bursting test (C) of blank kraft paper and coated paper samples.

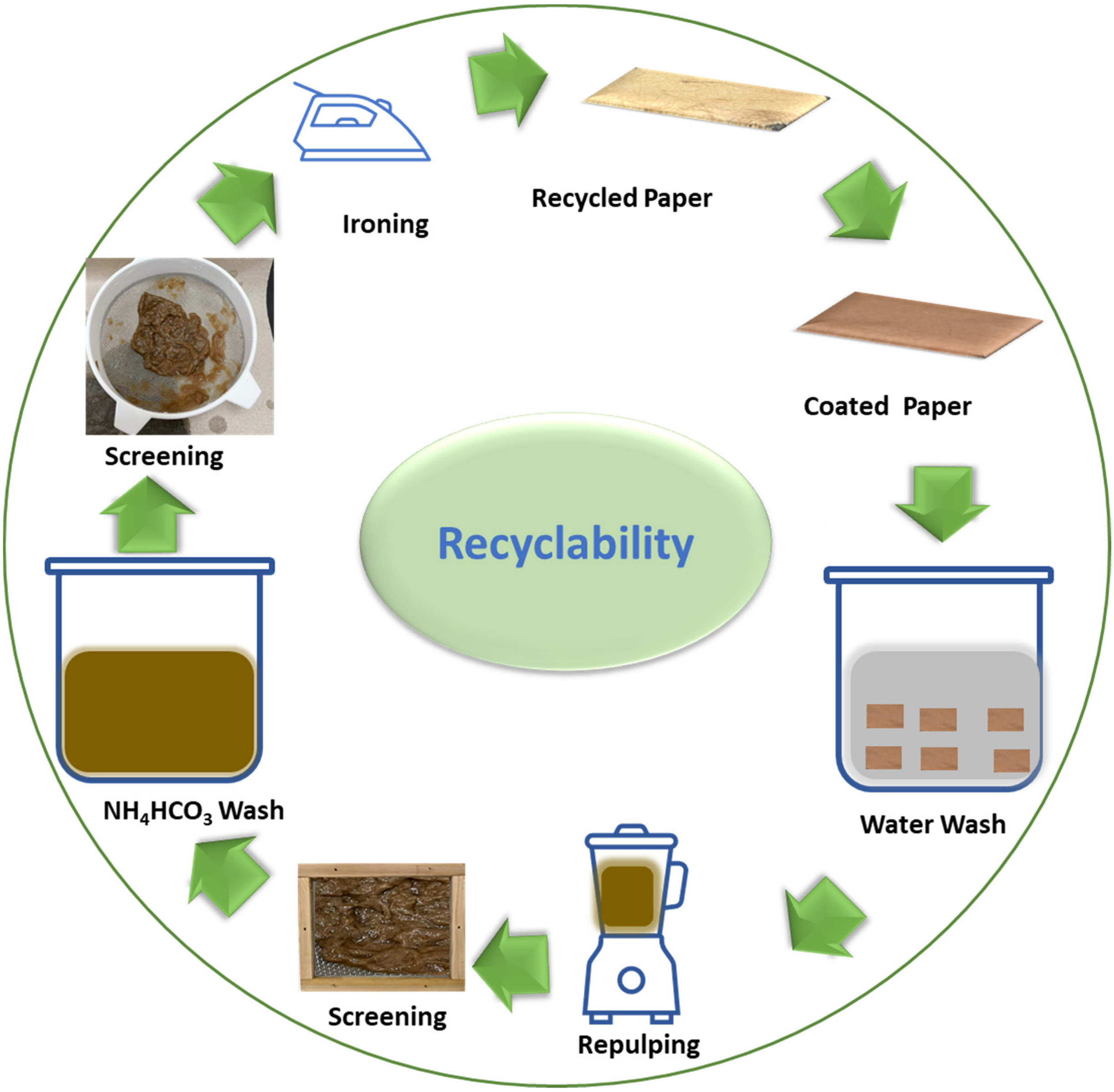

Scheme S2. Paper recycling via the repulping approach using blank kraft paper and P1S4-coated paper.

Statistical Analysis

Table S2. Tukey multiple mean comparisons of treatment effects on **water contact angle** after 5 min.

Difference of Levels	Difference of Means	SE of Difference	95% CI	T-Value	Adjusted P-Value
P1S0 - B-KP	87.65	1.13	(83.18, 92.12)	77.72	0.000
P1S1 - B-KP	80.26	1.13	(75.79, 84.73)	71.17	0.000
P2S1 - B-KP	76.05	1.13	(71.58, 80.52)	67.44	0.000
P3S1 - B-KP	87.55	1.13	(83.08, 92.02)	77.63	0.000
P4S1 - B-KP	81.80	1.13	(77.33, 86.27)	72.54	0.000
S-5 - B-KP	45.80	1.13	(41.33, 50.27)	40.61	0.000
P1S1 - P1S0	-7.39	1.13	(-11.86, -2.92)	-6.55	0.003
P2S1 - P1S0	-11.60	1.13	(-16.07, -7.13)	-10.29	0.000
P3S1 - P1S0	-0.10	1.13	(-4.57, 4.37)	-0.09	1.000
P4S1 - P1S0	-5.85	1.13	(-10.32, -1.38)	-5.19	0.013
S-5 - P1S0	-41.85	1.13	(-46.32, -37.38)	-37.11	0.000
P2S1 - P1S1	-4.21	1.13	(-8.68, 0.26)	-3.73	0.066
P3S1 - P1S1	7.29	1.13	(2.82, 11.76)	6.46	0.004
P4S1 - P1S1	1.54	1.13	(-2.93, 6.01)	1.37	0.804

S-5 - P1S1	-34.46	1.13	(-38.93, -29.99)	-30.56	0.000
P3S1 - P2S1	11.50	1.13	(7.03, 15.97)	10.20	0.000
P4S1 - P2S1	5.75	1.13	(1.28, 10.22)	5.10	0.014
S-5 - P2S1	-30.25	1.13	(-34.72, -25.78)	-26.82	0.000
P4S1 - P3S1	-5.75	1.13	(-10.22, -1.28)	-5.10	0.014
S-5 - P3S1	-41.75	1.13	(-46.22, -37.28)	-37.02	0.000
S-5 - P4S1	-36.00	1.13	(-40.47, -31.53)	-31.92	0.000

Individual confidence level = 99.46%.

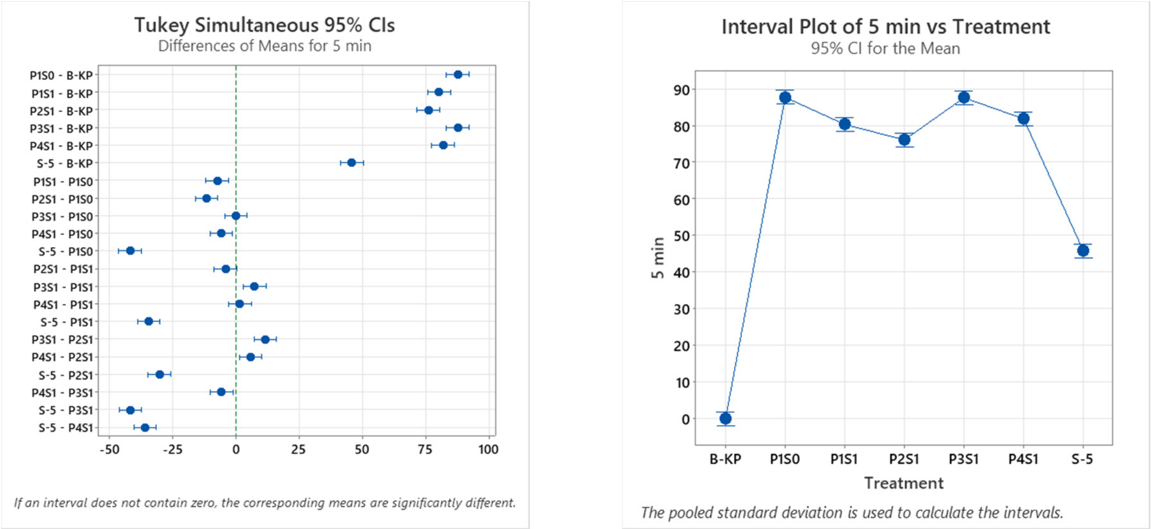

Figure S6. Effect of treatments on water contact angle after 5 min.

Table S3. Tukey multiple mean comparisons of treatment effects on oil contact angle after 5 min.

Difference of Levels	Difference of Means	SE of Difference	95% CI	T-Value	Adjusted P-Value
P1S0 - B-KP	43.55	1.82	(36.34, 50.76)	23.98	0.000
P1S1 - B-KP	30.45	1.82	(23.24, 37.66)	16.76	0.000
P2S1 - B-KP	36.60	1.82	(29.39, 43.81)	20.15	0.000
P3S1 - B-KP	37.50	1.82	(30.29, 44.71)	20.65	0.000
P4S1 - B-KP	42.20	1.82	(34.99, 49.41)	23.23	0.000
S-5 - B-KP	-8.35	1.82	(-15.56, -1.14)	-4.60	0.024
P1S1 - P1S0	-13.10	1.82	(-20.31, -5.89)	-7.21	0.002
P2S1 - P1S0	-6.95	1.82	(-14.16, 0.26)	-3.83	0.059
P3S1 - P1S0	-6.05	1.82	(-13.26, 1.16)	-3.33	0.106
P4S1 - P1S0	-1.35	1.82	(-8.56, 5.86)	-0.74	0.984
S-5 - P1S0	-51.90	1.82	(-59.11, -44.69)	-28.57	0.000
P2S1 - P1S1	6.15	1.82	(-1.06, 13.36)	3.39	0.099
P3S1 - P1S1	7.05	1.82	(-0.16, 14.26)	3.88	0.055
P4S1 - P1S1	11.75	1.82	(4.54, 18.96)	6.47	0.004
S-5 - P1S1	-38.80	1.82	(-46.01, -31.59)	-21.36	0.000
P3S1 - P2S1	0.90	1.82	(-6.31, 8.11)	0.50	0.998
P4S1 - P2S1	5.60	1.82	(-1.61, 12.81)	3.08	0.143
S-5 - P2S1	-44.95	1.82	(-52.16, -37.74)	-24.75	0.000
P4S1 - P3S1	4.70	1.82	(-2.51, 11.91)	2.59	0.257
S-5 - P3S1	-45.85	1.82	(-53.06, -38.64)	-25.24	0.000
S-5 - P4S1	-50.55	1.82	(-57.76, -43.34)	-27.83	0.000

Individual confidence level = 99.46%.

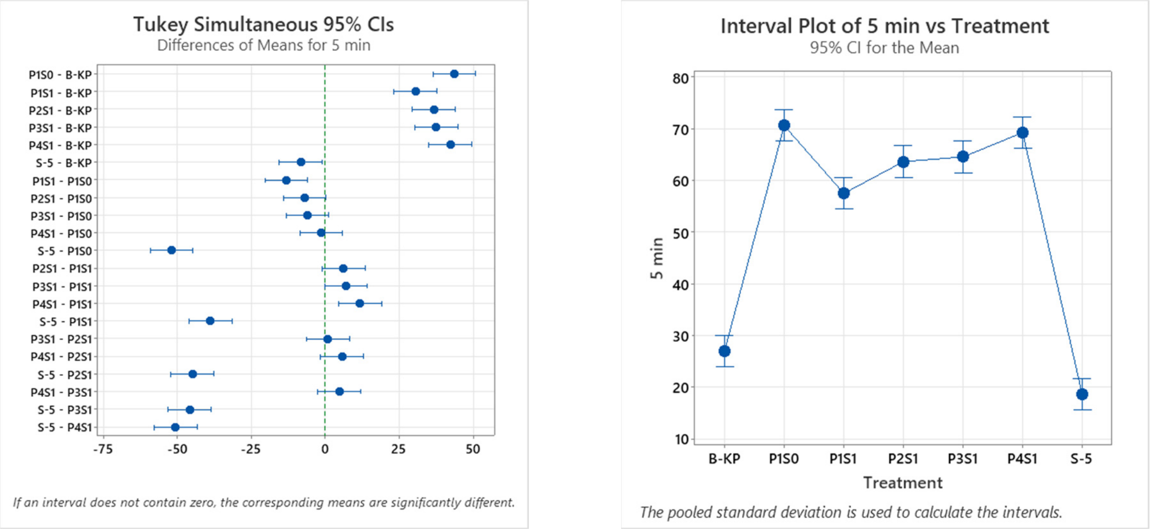

Figure S7. Effect of treatments on oil contact angle after 5 min.

Table S4. Tukey multiple mean comparisons of treatment effects on tensile strength (MD).

Difference of Levels	Difference of Means	SE of Difference	95% CI	T-Value	Adjusted P-Value
P1S0 - B-KP	4.87	1.34	(0.46, 9.28)	3.63	0.030
P2S1 - B-KP	-0.73	1.34	(-5.14, 3.68)	-0.55	0.980
P4S1 - B-KP	3.17	1.34	(-1.24, 7.58)	2.36	0.203
S-5 - B-KP	7.20	1.34	(2.79, 11.61)	5.37	0.002
P2S1 - P1S0	-5.60	1.34	(-10.01, -1.19)	-4.18	0.013
P4S1 - P1S0	-1.70	1.34	(-6.11, 2.71)	-1.27	0.715
S-5 - P1S0	2.33	1.34	(-2.08, 6.74)	1.74	0.454
P4S1 - P2S1	3.90	1.34	(-0.51, 8.31)	2.91	0.090
S-5 - P2S1	7.93	1.34	(3.52, 12.34)	5.92	0.001
S-5 - P4S1	4.03	1.34	(-0.38, 8.44)	3.01	0.078

Individual confidence level = 99.18%.

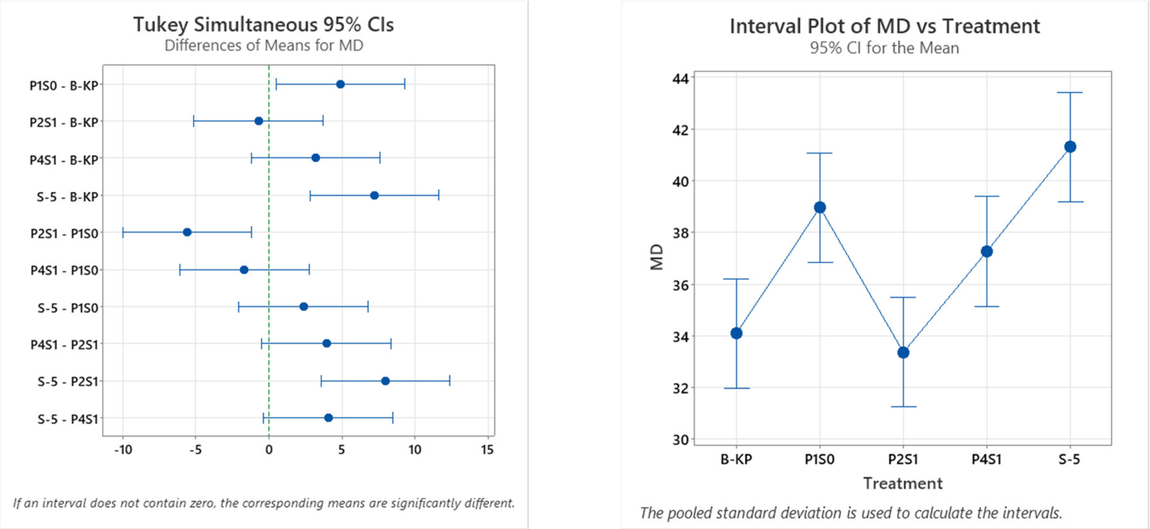

Figure S8. Effect of treatments on tensile strength (MD).

Table S5. Tukey multiple mean comparisons of treatment effects on tensile strength (CD).

Difference of Levels	Difference of Means	SE of Difference	95% CI	T-Value	Adjusted P-Value
P1S0 - B-KP	-1.800	0.552	(-3.616, 0.016)	-3.26	0.052
P2S1 - B-KP	-1.267	0.552	(-3.082, 0.549)	-2.29	0.223

P4S1 - B-KP	-1.400	0.552	(-3.216, 0.416)	-2.54	0.158
S-5 - B-KP	2.533	0.552	(0.718, 4.349)	4.59	0.007
P2S1 - P1S0	0.533	0.552	(-1.282, 2.349)	0.97	0.864
P4S1 - P1S0	0.400	0.552	(-1.416, 2.216)	0.72	0.946
S-5 - P1S0	4.333	0.552	(2.518, 6.149)	7.85	0.000
P4S1 - P2S1	-0.133	0.552	(-1.949, 1.682)	-0.24	0.999
S-5 - P2S1	3.800	0.552	(1.984, 5.616)	6.88	0.000
S-5 - P4S1	3.933	0.552	(2.118, 5.749)	7.12	0.000

Individual confidence level = 99.18%.

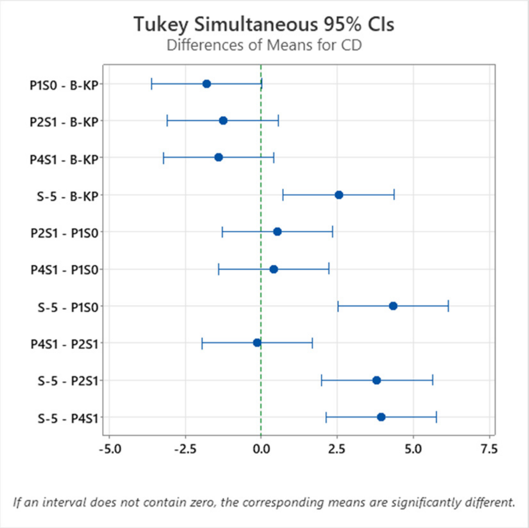

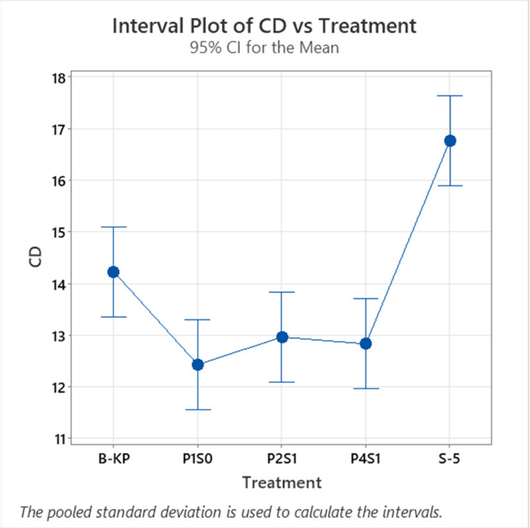

Figure S9. Effect of treatments on tensile strength (CD).

Table S6. Tukey multiple mean comparisons of treatment effects on % elongation (MD).

Difference of Levels	Difference of Means	SE of Difference	95% CI	T-Value	Adjusted P-Value
P1S0 - B-KP	0.593	0.125	(0.181, 1.006)	4.73	0.006
P2S1 - B-KP	0.773	0.125	(0.361, 1.186)	6.17	0.001
P4S1 - B-KP	0.757	0.125	(0.344, 1.169)	6.04	0.001
S-5 - B-KP	0.950	0.125	(0.538, 1.362)	7.58	0.000
P2S1 - P1S0	0.180	0.125	(-0.232, 0.592)	1.44	0.621
P4S1 - P1S0	0.163	0.125	(-0.249, 0.576)	1.30	0.696
S-5 - P1S0	0.357	0.125	(-0.056, 0.769)	2.84	0.099
P4S1 - P2S1	-0.017	0.125	(-0.429, 0.396)	-0.13	1.000
S-5 - P2S1	0.177	0.125	(-0.236, 0.589)	1.41	0.636
S-5 - P4S1	0.193	0.125	(-0.219, 0.606)	1.54	0.561

Individual confidence level = 99.18%.

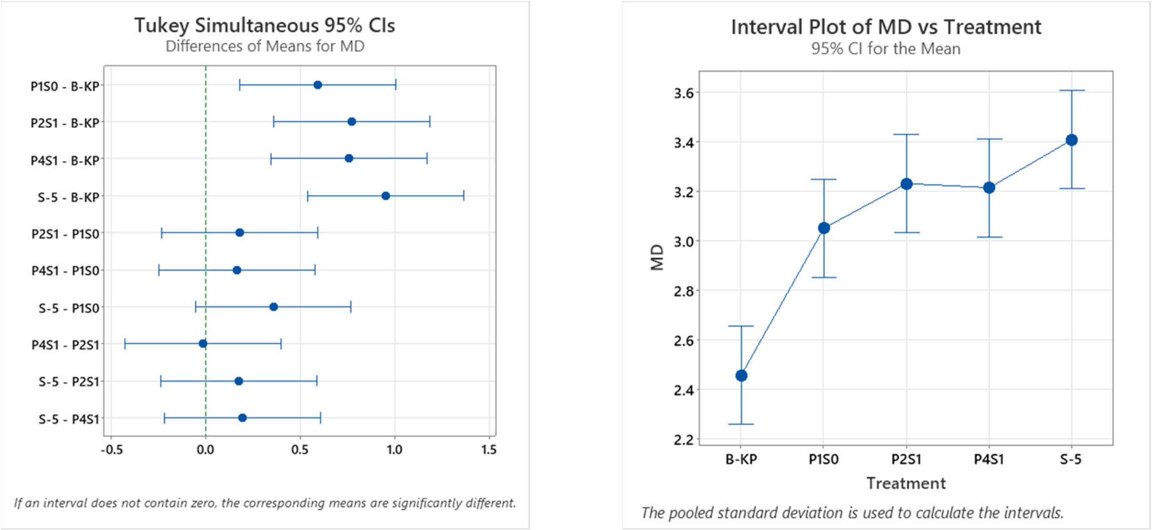

Figure S10. Effect of treatments on % elongation (MD).

Table S7. Tukey multiple mean comparisons of treatment effects on % elongation (CD).

Difference of Levels	Difference of Means	SE of Difference	95% CI	T-Value	Adjusted P-Value
P1S0 - B-KP	-0.710	0.478	(-2.280, 0.860)	-1.49	0.592
P2S1 - B-KP	0.057	0.478	(-1.514, 1.627)	0.12	1.000
P4S1 - B-KP	-0.513	0.478	(-2.084, 1.057)	-1.07	0.815
S-5 - B-KP	0.923	0.478	(-0.647, 2.494)	1.93	0.361
P2S1 - P1S0	0.767	0.478	(-0.804, 2.337)	1.61	0.526
P4S1 - P1S0	0.197	0.478	(-1.374, 1.767)	0.41	0.993
S-5 - P1S0	1.633	0.478	(0.063, 3.204)	3.42	0.041
P4S1 - P2S1	-0.570	0.478	(-2.140, 1.000)	-1.19	0.755
S-5 - P2S1	0.867	0.478	(-0.704, 2.437)	1.81	0.417
S-5 - P4S1	1.437	0.478	(-0.134, 3.007)	3.01	0.077

Individual confidence level = 99.18%.

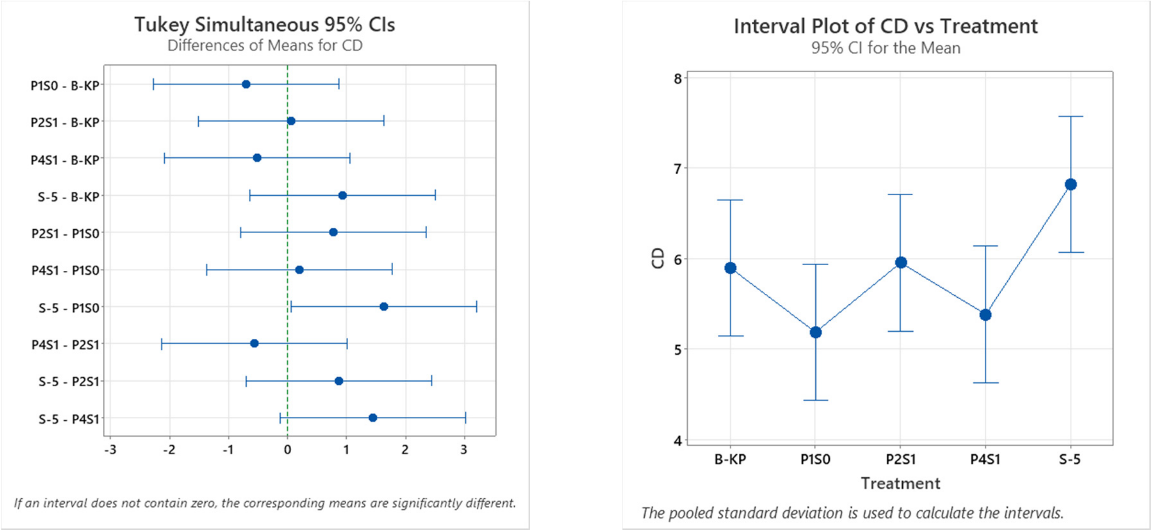

Figure S11. Effect of treatments on % elongation (CD).

Table S8. Tukey multiple mean comparisons of treatment effects on Young's modulus (MD).

Difference of Levels	Difference of Means	SE of Difference	95% CI	T-Value	Adjusted P-Value
P1S0 - B-KP	120	102	(-214, 454)	1.18	0.762
P2S1 - B-KP	-397	102	(-731, -62)	-3.90	0.019

P4S1 - B-KP	-97	102	(-431, 238)	-0.95	0.870
S-5 - B-KP	240	102	(-94, 574)	2.36	0.203
P2S1 - P1S0	-517	102	(-851, -182)	-5.08	0.003
P4S1 - P1S0	-217	102	(-551, 118)	-2.13	0.279
S-5 - P1S0	120	102	(-214, 454)	1.18	0.762
P4S1 - P2S1	300	102	(-34, 634)	2.95	0.085
S-5 - P2S1	637	102	(302, 971)	6.26	0.001
S-5 - P4S1	337	102	(2, 671)	3.31	0.048

Individual confidence level = 99.18%.

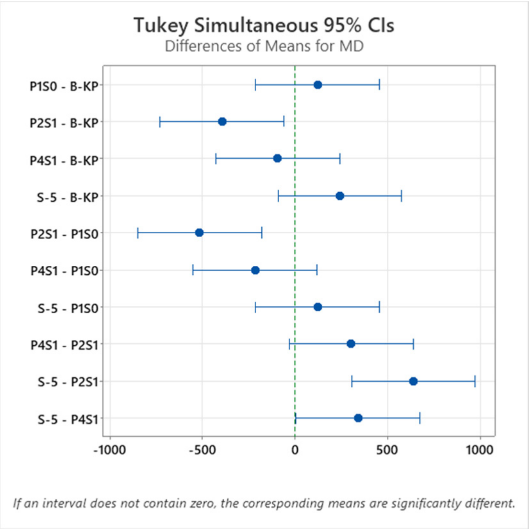

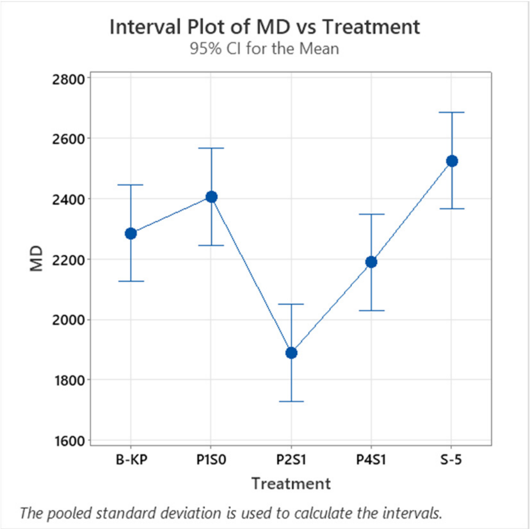

Figure S12. Effect of treatments on Young's modulus (MD).

Table S9. Tukey multiple mean comparisons of treatment effects on Young's modulus (CD).

Difference of Levels	Difference of Means	SE of Difference	95% CI	T-Value	Adjusted P-Value
P1S0 - B-KP	-327.3	71.0	(-560.7, -94.0)	-4.61	0.007
P2S1 - B-KP	-271.7	71.0	(-505.0, -38.3)	-3.83	0.022
P4S1 - B-KP	-301.7	71.0	(-535.0, -68.3)	-4.25	0.011
S-5 - B-KP	41.7	71.0	(-191.7, 275.0)	0.59	0.974
P2S1 - P1S0	55.7	71.0	(-177.7, 289.0)	0.78	0.929
P4S1 - P1S0	25.7	71.0	(-207.7, 259.0)	0.36	0.996
S-5 - P1S0	369.0	71.0	(135.7, 602.3)	5.20	0.003
P4S1 - P2S1	-30.0	71.0	(-263.3, 203.3)	-0.42	0.992
S-5 - P2S1	313.3	71.0	(80.0, 546.7)	4.42	0.009
S-5 - P4S1	343.3	71.0	(110.0, 576.7)	4.84	0.005

Individual confidence level = 99.18%.

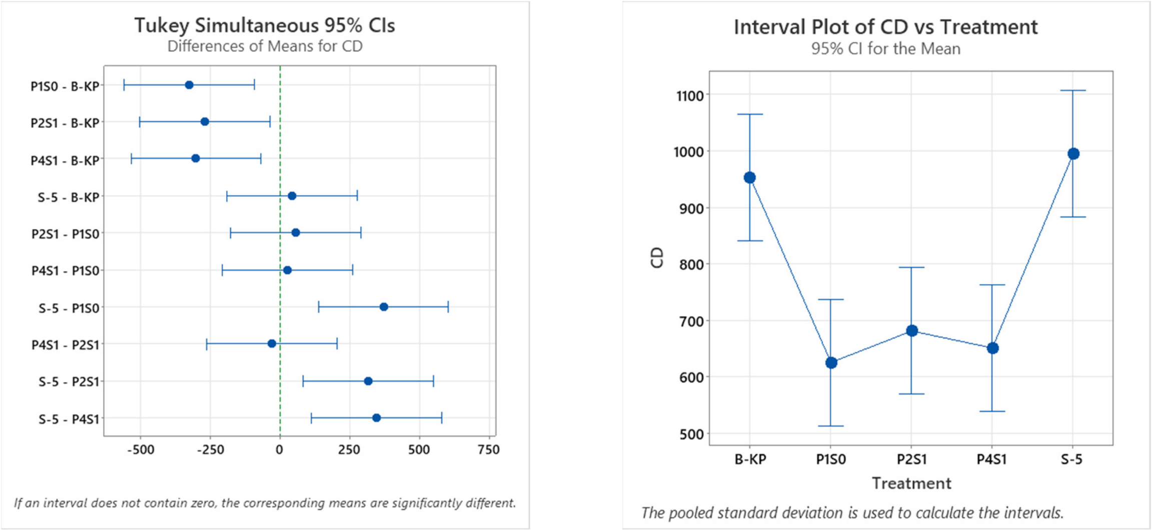

Figure S13. Effect of treatments on Young's modulus (CD).

Table S10. provides a summary of our previously reported work in comparison to our current work to provide a better understanding of our work.

Table S10. Summary of literature on paper coating approaches showing water and oil resistance coating.

Coating Materials	Method	Cobb60 g/m^2	Kit Rating	References
		**Present Work**		
**PDMS-Starch**	Dual layer approach using starch as a base layer and waterborne emulsion of PDMS, blending with starch.	2.7 ± 0.14	12	Present work
		Previous Work		
**PDMS-*grafted*-Chitosan**	Single layer, PDMS was grafted to chitosan and zein was used as a filler.	21	11	[1]
**Starch/Zein**	Dual layer; starch–zein-based coating. Using starch as a base layer and zein as a top layer.	6.2	12	[2]
**PVOH-Zein**	Dual layer; PVOH–zein-based coating. Using PVOH as a base layer and zein as a top layer.	3.3	12	[3]
**Chitosan/Zein**	Dual layer; chitosan–zein-based coating. Using chitosan as a base layer and zein as a top layer.	4.88	12	[4]

Cost analysis for P1S1, where PDMS-COOH and starch are at 50:50 wt% ratios:

For P1S1, where PDMS-COOH and starch are at 50:50 wt% ratios:

Cost of PDMS and Dianhydride Mixture (where each $ is reported in USD):

Since PDMS is prepared by reacting ~95 wt% PDMS with ~5 wt% dianhydride; therefore, for one 1 kg of PDMS-COOH:

The cost of PDMS = 0.95 kg × $10/kg = $9.50 (assuming PDMS cost $10/kg based on conversation with WACKER Silicone)

The cost of dianhydride = 0.05 kg × $20/kg = $1.00 (assuming dianhydride cost $20/kg, based on publicly available data)

The cost for 1 kg of PDMS-COOH = $9.50 + $1.00 = **$10.50 per kg**

Cost for the Final Composition:

The final material is 50% PDMS-COOH and 50% starch.

Cost for 0.5 kg of PDMS-COOH = $10.50 × 0.5 = $5.25

Cost for 0.5 kg of Starch = $0.40 × 0.5 = $0.20 (Assuming starch price as 40 cents per Kg, based on online publicly available data)

Total cost for 1 kg of the final material = $5.25 + $0.20 = $5.45

Therefore, the cost per kg of the final material, which is a blend of PDMS-COOH, and starch in the specified ratios, is $5.45 per kg. This cost in the range of polymers such as polyhydroxyalkanoates (PHAs), poly(butylene adipate-*co*-terephthalate) (PBAT), etc.

References

1. Hamdani, S.S.; Li, Z.; Rabnawaz, M.; Kamdem, D.P.; Khan, B.A., Chitosan–Graft–Poly(dimethylsiloxane)/Zein Coatings for the Fabrication of Environmentally Friendly Oil- and Water-Resistant Paper. *ACS Sustainable Chem. Eng.* **2020,** *8* (13), 5147-5155.
2. Kansal, D.; Hamdani, S.S.; Ping, R.; Rabnawaz, M., Starch and Zein Biopolymers as a Sustainable Replacement for PFAS, Silicone Oil, and Plastic-Coated Paper. *Ind. Eng. Chem. Res.* **2020,** *59* (26), 12075-12084.
3. Hamdani, S.S.; Li, Z.; Sirinakbumrung, N.; Rabnawaz, M., Zein and PVOH-Based Bilayer Approach for Plastic-Free, Repulpable and Biodegradable Oil- and Water-Resistant Paper as a Replacement for Single-Use Plastics. *Ind. Eng. Chem. Res.* **2020,** *59* (40), 17856-17866.
4. Kansal, D.; Hamdani, S.S.; Ping, R.; Sirinakbumrung, N.; Rabnawaz, M., Food-Safe Chitosan–Zein Dual-Layer Coating for Water- and Oil-Repellent Paper Substrates. *ACS Sustainable Chem. Eng.* **2020,** *8* (17), 6887-6897.

Disclaimer/Publisher's Note: The statements, opinions and data contained in all publications are solely those of the individual author(s) and contributor(s) and not of MDPI and/or the editor(s). MDPI and/or the editor(s) disclaim responsibility for any injury to people or property resulting from any ideas, methods, instructions or products referred to in the content.
